# Spatial Clustering for Carolina Breast Cancer Study

**DOI:** 10.1142/9789819807024_0025

**Published:** 2025

**Authors:** Hongqian Niu, Melissa Troester, Didong Li

**Affiliations:** 1 Department of Biostatistics, University of North Carolina, Chapel Hill, NC, USA; 2 Department of Epidemiology, University of North Carolina, Chapel Hill, NC, USA

**Keywords:** Census tracts, Gaussian process, Socioeconomic status

## Abstract

In the Carolina Breast Cancer Study (CBCS), clustering census tracts based on spatial location, demographic variables, and socioeconomic status is crucial for understanding how these factors influence health outcomes and cancer risk. This task, known as spatial clustering, involves identifying clusters of similar locations by considering both geographic and characteristic patterns. While standard clustering methods such as K-means, spectral clustering, and hierarchical clustering are well-studied, spatial clustering is less explored due to the inherent differences between spatial domains and their corresponding covariates. In this paper, we introduce a spatial clustering algorithm called Gaussian Process Spatial Clustering (GPSC). GPSC leverages the flexibility of Gaussian Processes to cluster unobserved functions between different domains, extending traditional clustering techniques to effectively handle geospatial data. We provide theoretical guarantees for GPSC’s performance and demonstrate its capability to recover true clusters through several empirical studies. Specifically, we identify clusters of census tracts in North Carolina based on socioeconomic and environmental indicators associated with health and cancer risk.

## 1. Introduction

There is growing research suggesting that socioenvironmental factors can play a key role in affecting health outcomes, potentially contributing to health disparities in marginalized groups, and may even predictably impact outcomes at the molecular level with diseases such as cancer.^1,2^ However, identifying areas of such risk can be a difficult task. In the community-wide socioeconomic and environmental indicators dataset, the spatial locations of North Carolina census tracts were paired with socioeconomic data from the American Community Survey^3^ from 2014 chosen to reflect socioeconomic advantage and disadvantage,^4^ as well as environmental pollution data from the U.S. Environmental Protection Agency (EPA) National Air Toxics Assessment (NATA^2,5^). This then poses the problem: how can geographically spread NC census tracts be clustered together based on risk factors including socioeconomic indicators and environmental pollution? North Carolina is known to be an ethnically diverse state,^6^ with a wide range of spatially dependent differences in socioeconomic status such as access to healthcare, poverty rates, and education, while meaningful clusterings must take into consideration all these differences.^6^ A standard clustering algorithm applied to the data collected from the patients in each tract or to the environmental variables alone fails to necessarily capture the significant spatial dependence inherent in the data collected in the studies. This problem is known as spatial clustering or geospatial clustering.^7^

In spatial clustering, the goal is to identify clusters of similar locations based on regionalization, as well as patterns in characteristics over those locations. Clustering of geospatial data is a common unsupervised learning problem with many applications to areas, e.g., public health,^8^ urban planning,^9^ or transportation,^10^ where geography plays an essential role.

Furthermore, spatial data, also known as geospatial data, is commonly characterized by having a distinct geographic component.^11^ Unlike traditional data that only include observations as a single set of features x, spatial data may be considered as a vector [s,x], where $s\in {\mathbb{R}}^{2}$ represents the spatial location of the observation and $x\in {\mathbb{R}}^{p}$ is the set of features or covariates. The analysis of such spatial datasets poses challenges, such as accurately capturing the relative effects between the spatial and covariate domains.^11^ Importantly, geographically close areas may still have very different patterns of characteristics, while separated areas may share similarities and constitute a single functional cluster. Together, this can pose challenges to traditional clustering methods that equally treat the separate domains inherent to geospatial data such as K-means, as the geographic locations of distinct clusters may be well mixed, or the measurements themselves of different variables at those locations may be well mixed.

Without the spatial component, clustering itself is a well-studied problem with many established techniques such as K-means clustering,^12^ spectral clustering,^13^ hierarchical clustering,^14^ and density-based spatial clustering of applications with noise (DBSCAN^15^), to name a few popular algorithms. Each of these algorithms offers distinct advantages based on their modeling assumptions when performed on different types of data. Additionally, common extensions of these algorithms include supervised fuzzy C-means,^16^ spatial hierarchical clustering,^17^ and the generalized DBSCAN (GDBSCAN^18^) algorithm. These algorithms are able to better incorporate either response labels or spatial data directly through customized distance metrics or connectivity constraints.

However, in this paper, we consider the case of supervised spatial data, with observations consisting of three components (s,x,y), where $s\in {\mathbb{R}}^{2}$ is the spatial component, $x\in {\mathbb{R}}^{p}$ is the feature component, while y∈ℝ is the response variable of particular interests. Assuming that in the data there is a relationship between features x, or between features and geography (s,x), and the response y, we propose a new spatial clustering algorithm based on Gaussian Processes (GPs), called Gaussian Process Spatial Clustering (GPSC), which groups together clusters based on each group’s ability to predict the response variable y. We focus on single-output cases in this paper for simplicity, but the extension to multi-output cases where $y\in {\mathbb{R}}^{d}$ with d>1 is straightforward.

For the motivating example from NC census tracts data, *s* is the longitude/latitude pairs defining each state census tract, x is the set of environmental pollution variables such as levels of hexane, lead, mercury, etc, as well as average socioeconomic indicators such as unemployment rates, poverty rates, or education, and the *y* response to be predicted is a previously defined latent class^2^ measuring socioeconomic and environmental advantage-disadvantage.

**Fig. 1:**
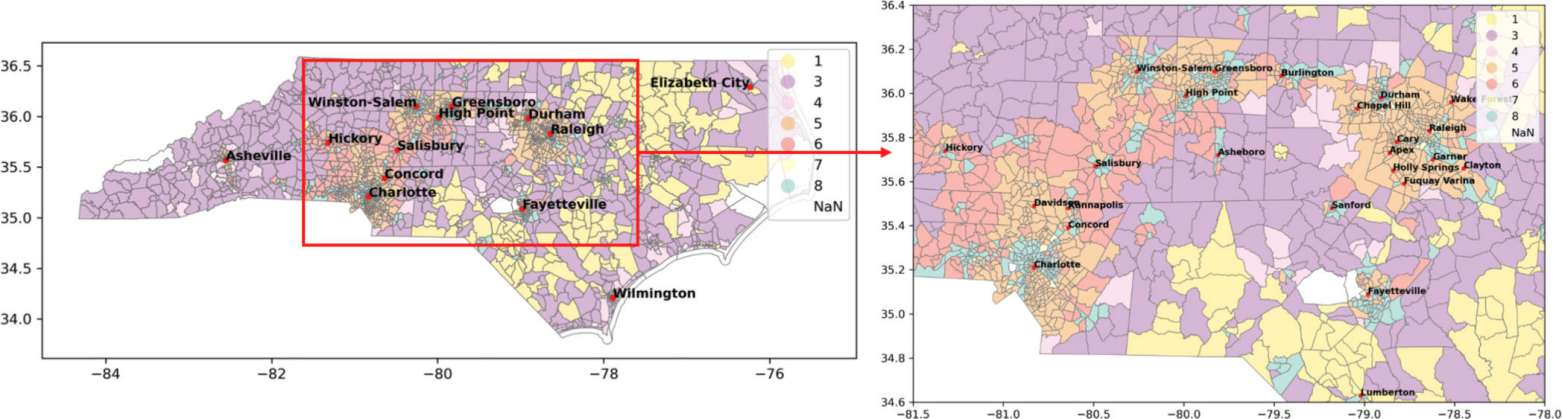
Socioeconomic and environmental advantage-disadvantage latent class map of NC.

In order to do so, GPSC leverages the flexibility of GPs, well-studied near-universal function approximators,^19,20^ to fit the true functional relationships within each clustering and to cluster tract locations and features pertaining to socioeconomic status. Simulation studies show that the GPSC algorithm is capable of accurately recovering and clustering these functional relationships even in cases of limited spatial dependencies such as in the case of irregular cluster shapes or sizes, and regardless of any dependencies in the covariate domain. This is important because, as in Figure 1, clusters may not always be completely separated, so it is essential to control the relative influence of each domain in the clustering done in GPSC by choosing the kernel. Furthermore, GPSC is less sensitive to dependencies in the covariate domain compared to traditional clustering methods such as K-means clustering. We prove that GPSC is able to find the true clusters as long as the functional relationships between the clusters are distinct. When applied to community-wide study, GPSC successfully clusters tracts in NC with finer detail than traditional methods and can be interpreted by domain experts.

In summary, our contributions in this paper are 1) a novel spatial clustering GPSC algorithm, 2) theoretical support to GPSC and 3) application to NC tract level data with new interpretable discoveries. Full proofs of theorems, implementation details, as well as extended simulations are presented in the Supplementary Material at https://github.com/hong-niu/gpsc-psb25.

## 2. Model

### 2.1. Gaussian Process Regression

In this section, we review the GP model and its application towards regression and classification. By definition, a GP is a random function for which any finite realization follows a multivariate Gaussian distribution:^21^

#### Definition 2.1.

f follows GP in domain ! with mean function μ and covariance function K, denoted by f∼GP(μ,K), where μ:Ω→ℝ, K:Ω×Ω→ℝ, if for any $x_{1},\cdots ,x_{n}\in \text{Ω}$,

$${\left[y_{1},\cdots ,y_{n}\right]}^{⊤}:={\left[f\left(x_{1}\right),\cdots ,f\left(x_{n}\right)\right]}^{⊤}∼N(v,\text{Σ}),$$

where $v={\left[\mu \left(x_{1}\right),\cdots ,\mu \left(x_{n}\right)\right]}^{⊤}$ and ${\text{Σ}}_{ij}=K\left(x_{i},x_{j}\right)$.

A GP is completely determined by the mean function μ and the covariance function K, also known as the kernel. In this paper, we assume μ=0 for simplicity and use the radial basis function (RBF), also known as the squared exponential kernel, defined as: $K\left(x,x^{\prime }\right)=\sigma^{2}e^{-\frac{d^{2}\left(x,x^{\prime }\right)}{2b}}$, but our model can be extended to other kernels. The two parameters, i.e., spatial variance $\sigma^{2}$ and length scale b are estimated by maximizing the likelihood (MLE). Given training data ${\left(x_{i},y_{i}\right)}_{i=1}^{n}$ with MLE $\theta_{n}=\left(\sigma_{n}^{2},b_{n}\right)$ and a new observation $x_{*}$, the best unbiased linear predictor (BLUP^22^) of $y_{*}=f\left(x_{*}\right)$ is given by ${\hat{y}}_{*}=K_{\theta_{n}}\left(x_{*},X\right)K_{\theta_{n}}{\left(X,X\right)}^{-1}Y$, where $K_{\theta_{n}}{\left(x_{*},X\right)}_{i}=K_{\theta_{n}}\left(x_{*},x_{i}\right)$, $K_{\theta_{n}}{\left(X,X\right)}_{ij}=K_{\theta_{n}}\left(x_{i},x_{j}\right)$ and $Y={\left[y_{1},\cdots ,y_{n}\right]}^{⊤}\in {\mathbb{R}}^{n}$ Rn. As a flexible regression algorithm, GP can be modified into a classifier using a link function^21^ for a discrete response variable y, so we will not distinguish between Gaussian process regression (GPR) and Gaussian process classification (GPC) in this paper.

### 2.2. GP Spatial Clustering

Now we will consider observations ${\left\{\left(s_{i},x_{i},y_{i}\right)\right\}}_{i=1}^{n}$, where $s_{i}\in S\subset {\mathbb{R}}^{2}$ is the spatial location, $x_{i}\in \text{Ω}\in {\mathbb{R}}^{p}$ is the covariate, and $y_{i}$ is the response variable. Let $l_{i}\in \{1,\cdots ,L\}$ be the unobserved cluster label such that $l_{i}=j\Leftrightarrow s_{i}\in S_{j}\subset S$^,^ where $S_{1},\cdots ,S_{L}$ is a partition of Ω. We focus on the following model. $y_{i}=\sum_{j=1}^{L}1_{\left\{s_{i}\in S_{i}\right\}}f_{j}\left(x_{i}\right)=\sum_{j=1}^{L}1_{\left\{l_{i}=j\right\}}f_{j}\left(x_{i}\right)$, where $f_{j}$ is unknown function on Ω in certain function class that will be discussed in Section 3. That is, the functional relation between $y_{i}$ and $x_{i}$ varies across spatial clusters supported by $S_{i}$. The goal is to recover the cluster label $l_{i}$, called spatial clustering since the clusters are rooted in the spatial domain S.

For example, in the NC tracts data, each $S_{i}$ consists of tracts in NC, while the relationship between the latent class and the socioeconomic and environmental covariates varies across the tracts spatially. The goal is to partition NC into several clusters so that each cluster admits a unique functional relationship.

For a given observation $x_{i}$ in cluster j with response $y_{i}$, we expect the prediction error of $f_{j}$ to be the lowest among all $f_{j}$ ‘s, and hence we can assign $x_{i}$ to the cluster with the lowest prediction error. However, neither the cluster label $l_{i}$ or domain partition $S_{i}$, nor the functions $f_{j}$ is observed. Motivated by the flexibility of GP models, we use GP to approximate the unobserved functions $f_{j}$, denoted by $\hat{f_{j}}$, and assign $x_{i}$ to the cluster labeled by $\hat{l_{i}}$ with the lowest prediction error: ${\hat{l}}_{i}=\text{argmi}{\text{n}}_{j}{\left({\hat{f}}_{j}\left(s_{i},x_{i}\right)-y_{i}\right)}^{2}$. Then we update the cluster and $\hat{f_{j}}$ iteratively. The GPSC algorithm is summarized in algorithm 1.

**Algorithm 1:** Gaussian Process Spatial Clustering

Input: data ${\left(s_{i},x_{i},y_{i}\right)}_{i=1}^{n}$, number of clusters L, maximum number of iterations T Initialize ${\hat{l}}_{i}=\text{randomInt}(1,2,\cdots ,L)$ **for** t=1 **to** T **do** **for** j=1 **to** L **do** $\left(S_{j},X_{j},Y_{j}\right)=\left\{\left(s_{i},x_{i},y_{i}\right):{\hat{l}}_{i}=j\right\},{\hat{f}}_{j}=\mathrm{GPR}\left(\left(\left[S_{j},X_{j}\right],Y_{j}\right)\right)$ **end for** **for** i=1 **to** n **do** ${\hat{l}}_{i}=argmin_{j}{\left({\hat{f}}_{j}\left(\left(s_{i},x_{i}\right)\right)-y_{i}\right)}^{2}$ **end for** **end for**

In this flexible construction, it is also possible to extend the reassignment function for different applications, such as reinforcing spatial contiguity constraints as is common in geographical clustering:

$${\hat{l}}_{i}=\underset{j=1,\cdots ,L}{\mathrm{argmin}}\left\{{\left({\hat{f}}_{j}\left(s_{i},x_{i}\right)-y_{i}\right)}^{2}+\lambda \left\|s_{i}-C_{j}\right\|\right\}.$$

Here, $C_{j}$ is the center in the spatial domain of the current cluster $S_{j}$, while λ is a tuning parameter that controls the penalization of assigning points to clusters that are spatially distant. For the rest of the paper, we will focus on the case λ=0, but will demonstrate the effects of adding such penalties in the simulation studies.

In summary, the inputs to the algorithm are observations ${\left\{\left(s_{i},x_{i},y_{i}\right)\right\}}_{i=1}^{n}$, along with tuning parameters including the number of iterations T and the number of clusters L. In practice the number of iterations T need not necessarily be large, and can be replaced with the stopping criterion when the cluster assignments stabilize. The proper choice of the number of clusters L is a typical challenge in the field of clustering,^23^ which is beyond the scope of this paper. The choice of L often requires domain expertise specific to the application at hand, see Section 5 for more detailed discussion. In practice, we also typically bound the parameters of the covariance function during optimization to prevent overfitting.

## 3. Theory

In this section, we provide theoretical support to the GPSC algorithm. We start with the necessary definitions to state the assumptions and theorems.

### Definition 3.1.

Let K be a positive definite kernel on $\Omega \subset {\mathbb{R}}^{p}$, then $F_{K}(\Omega ):=\mathrm{span}\{K(\cdot ,x):x\in \Omega \}$ with inner product form ${\left(\sum_{i=1}^{n}a_{i}K\left(\cdot ,x_{i}\right),\sum_{j=1}^{m}b_{j}K\left(\cdot ,\;{\tilde{x}}_{j}\right)\right)}_{K}:=\sum_{i,j}a_{i}b_{j}K\left(x_{i},{\tilde{x}}_{j}\right)$, so that $F_{K}(\text{Ω})$ is a pre-Hilbert space with a reproducing kernel K. The linear mapping $\text{Φ}:F_{K}(\text{Ω})\to C(\text{Ω}):\text{Φ}(f)(x):={\left(f,K(\cdot ,x)\right)}_{K}$, is injective. Then the image of Φ, $N_{K}(\text{Ω}):=\text{Φ}\left(F_{K}(\text{Ω})\right)$ is a Hilbert space with a reproducing kernel K equipped with the inner product ${\left(f,g\right)}_{K}:={\left({\text{Φ}}^{-1}f,{\text{Φ}}^{-1}g\right)}_{K}$.

For simplicity, we fix $K_{\theta }$ to be the RBF kernel with $\theta =\left(\sigma^{2},b\right)$ from now on.

### Definition 3.2.

Given observations X and $x_{0}$ with unobserved $y_{0}$ to be predicted. Let $\psi_{X,x_{0}}:Y\mapsto K_{\theta (Y)}{\left(x_{0},X\right)}^{⊤}K_{\theta (Y)}{\left(X,X\right)}^{-1}Y$, where $\theta (Y)=\text{argma}{\text{x}}_{\theta }N(Y|0,K(X,X))$ is the maximum likelihood estimator of θ based on potential observations Y. That is, ψ is the BLUP of $y_{0}$ based on observations (X,Y). By the definition of ψ, the smoothness of the Gaussian density function and the linearity of BLUP, ψ is differentiable.^22^ We also introduce the following assumptions:

(A1) $\text{Ω}\subset {\mathbb{R}}^{p}$ is compact and p(x)>0, ∀x∈Ω, where p(x) is the density function of x.

(A2) $f_{j}\in N_{K}(\text{Ω})$, j=1,⋯,L.

### Theorem 3.3.

Under assumptions (A1)-(A2), at any iteration in Algorithm 1, let $n_{jk}:=\left|\left\{i:l_{i}=j,\hat{l_{i}}=k\right\}\right|$, $n_{j}:=\left|\left\{i:\hat{l_{i}}=j\right\}\right|$ then the current $x_{i}$ is a assigned to the correct cluster if for any k≠j,

(1) $$\frac{\sum_{m\neq j}n_{mj}}{\sum_{m\neq j}n_{mk}}<\frac{D_{l}E_{l}}{D_{u}E_{u}}-\frac{\|f{\|}_{K}e^{-c_{1}n_{j}^{\frac{1}{p}}}+\|f{\|}_{K}e^{-c_{2}n_{k}^{\frac{1}{p}}}}{D_{u}E_{u}n_{22}},$$

where $c_{1}$ and $c_{2}$ are constants, and

$$\begin{array}{l} D_{l}:=\inf\|\nabla \psi (Y)\|\leq D_{u}:=\|\nabla \psi (Y){\|}_{\infty },\\ E_{l}:=\underset{x\in \Omega ,j,k=1,\cdots ,L}{\inf}\left|f_{j}(x)-f_{k}(x)\right|\leq E_{u}:=\underset{x\in \Omega ,j,k=1,\cdots ,L}{\sup}\left|f_{j}(x)-f_{k}(x)\right|<\infty . \end{array}$$

In particular, let L=2, j=1, k=2 and let $n_{1}$,$n_{2}\to \infty$, Equation (1) becomes: $\frac{n_{21}}{n_{22}}<\frac{D_{l}E_{l}}{D_{u}E_{u}}$ That is, the mis-clustered proportion is small enough.

The right-hand side of inequality (1) is highly interpretable. The ratio $\frac{D_{l}}{D_{u}}$- measures the robustness of the BLUP, that is, how the BLUP changes with training data Y. The less robust the BLUP, the smaller the ratio, and the harder it is to find the correct clusters. The ratio $\frac{E_{l}}{E_{u}}$ measures the separation between functions $f_{1},\cdots ,f_{L}$. The smaller the separation, the smaller the ratio, and the harder it is to find the correct clusters. Theorem 3.3 also implies that the state of correct clustering is an absorbing state, that is, if the current clusters are close enough to the true clusters, then perfect clustering results will be achieved in the next iteration. Note that even if the inequality does not hold, the algorithm may still converge to a better state with more correctly clustered data, although not within one single step. This is because even when the right-hand side of Equation (1) is small, there might be some region ${\text{Ω}}_{0}\subset \text{Ω}$ where the $f_{j}$’s are relatively well separated so that the right-hand side is relatively large on ${\text{Ω}}_{0}$, so that samples within ${\text{Ω}}_{0}$ will be assigned to true clusters. Meanwhile, for the region where $f_{j}$’s are well mixed, it is challenging for all clustering algorithms.

In practice, the response variable y is often subject to measurement error, leading to a more realistic model: y=f(x)+ϵ, where $\epsilon ∼N\left(0,\tau^{2}\right)$ represents noise. The following theorem serves as the counterpart to Theorem 3.3 in the presence of Gaussian noise:

### Theorem 3.4.

Under the same assumption and notation as of Theorem 3.3, with the addition of Gaussian noise, the current $x_{i}$ is assigned to the correct cluster if for any k≠j,

(2) $$\frac{\sum_{m\neq j}n_{mj}}{\sum_{m\neq j}n_{mk}}<\frac{D_{l}E_{l}}{D_{u}E_{u}}-\frac{\|f{\|}_{K}e^{-c_{1}n_{j}^{\frac{1}{p}}}+\|f{\|}_{K}e^{-c_{2}n_{k}^{\frac{1}{p}}}+\xi }{D_{u}E_{u}n_{22}},$$

where ξ is the sum of independent χ-distributions with degrees of freedom 1, $n_{1}$ and $n_{2}$ rescaled by 2τ, τ and τ respectively.

In particular, when L=2, j=1, k=2, and $n_{1}$, $n_{2}\to \infty$, the right-hand side simplifies to $\frac{D_{l}E_{l}}{D_{u}E_{u}}$ with probability one. When τ=0, that is, the noise vanishes, then ξ=0 so Theorem 3.4 coincides with Theorem 3.3.

## 4. Simulation Studies

To evaluate the performance of GPSC, we present three simulation studies in this section, with detailed implementation details in the Supplementary Materials. The first simulation will demonstrate an application of Algorithm 1 in the case of responses generated by linear functions with two clusters, while the second simulation shows the performance of GPSC in the case of responses generated by nonlinear functions. The third simulation shows the robustness of GPSC to noisy data and over-specified number of clusters. In all simulations, we compare the performance of GPSC with traditional clustering algorithms: K-means, spectral clustering, hierarchical clustering, and DBSCAN, as well as spatial or supervised analogs: supervised fuzzy C-means, spatial hierarchical clustering, generalized GDBSCAN, and also the Gaussian mixture model (GMM^24^). We evaluate the performance using the adjusted Rand index (ARI^25^) and adjusted mutual information (AMI^26^) against the true labels. The data used in these simulations take the form ${\left\{\left(s_{i},x_{i},y_{i}\right)\right\}}_{i=1}^{n}$, where $s_{i}\in {\mathbb{R}}^{2}$ is the spatial domain, $x_{i}\in {\mathbb{R}}^{2}$ is the covariate domain, and $y_{i}\in \mathbb{R}$ is the response domain, taken for visualization purposes. Note that for all algorithms, including GPSC and the aforementioned traditional, nonspatial clustering algorithms, the input is taken to be the full vector (s,x,y) with the spatial domain included, so that all competitors always use the full information. The results can be directly extended to higher *p* and multivariate responses.

### 4.1. Simulation 1 - Linear Functions

In this simulation, *y* is a linear function of *x* for visualization purposes, where both *s*i and *x*i are generated from independent uniform distributions. After generating the data ${\left\{\left(s_{i},x_{i}\right)\right\}}_{i=1}^{n}$, the spatial domain is subdivided into two clusters, the center ball and the background region. The $y_{i}\in \mathbb{R}$ are then generated as distinct linear functions of $x_{i}$ for each cluster. For visualizations of the resulting clusters in the XY domain and all ARI/AMI scores, see Supplement D.1.

**Fig. 2:**
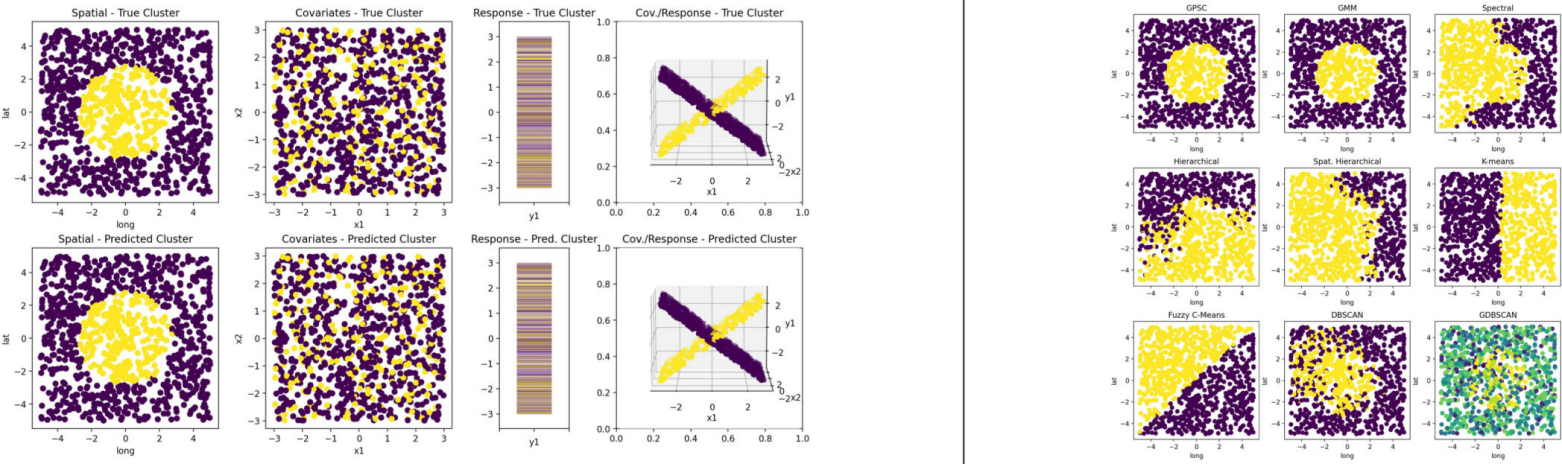
[Left] GPSC results for Simulation 1, colored by cluster. The first column plots the spatial domain $s_{i}\in {\mathbb{R}}^{2}$, the second column plots the covariate space $x_{i}\in {\mathbb{R}}^{2}$, the third column plots the response space $y_{i}\in \mathbb{R}$, while the right-most column plots $y_{i}\in \mathbb{R}$ against $x_{i}\in {\mathbb{R}}^{2}$. The first row shows the ground truth generated data. The second row shows the predicted clusters from GPSC after randomized initialization. [Right] Clusters for Simulation 1 by nine clustering algorithms visualized in the spatial domain.

It can be seen that this simulation is challenging for several reasons. First, there is almost no separation considering any dimension s*, x*, or y on its own as in the first three columns in Figure 2 (left); the separation is solely in the functional domain XY. As a result, most traditional algorithms cannot capture this functional relationship, as supported by Panels 3–7 in Figure 2 (right). Although it can seen that the Gaussian mixture model is able to rediscover the clusters in this case (Panel 2), this is due to GMM’s ability to estimate the pairwise linear correlation between each domain. However, we expect GMM to fail to capture nonlinear functional relationships, as shown in the following Simulation 2. It is also noted that DBSCAN and GDBSCAN (Panels 8 and 9) also perform reasonably well, but have challenges of their own such as GDBSCAN greatly overestimating the number of clusters.

### 4.2. Simulation 2 - Nonlinear Functions

**Fig. 3:**
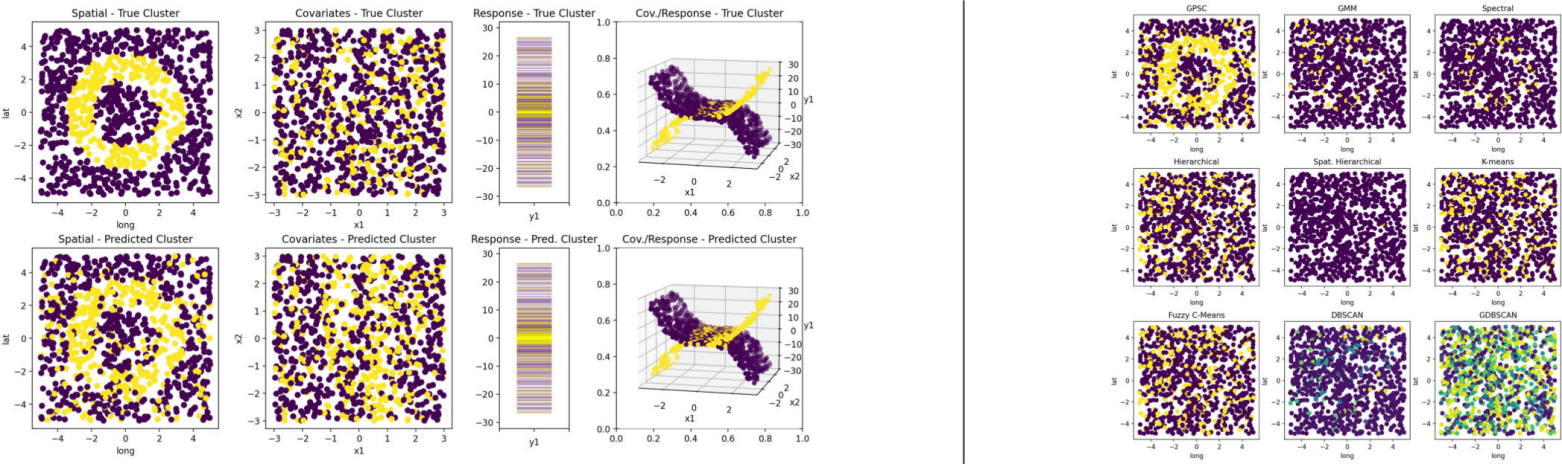
[Left] Results for Simulation 2 with true generated data (top) and results of GPSC (bottom). [Right] Clusters by nine different algorithms visualized in the spatial domain.

In this simulation, we will show that in an irregular spatial distribution with nonlinear relationships between the covariates and the response variable, GPSC is still able to recover the true functional relationships in contrast to the competitors. After generating the data ${\left\{\left(s_{i},x_{i}\right)\right\}}_{i=1}^{n}$ from independent uniform distributions, the spatial domain is subdivided into two clusters, the ring and the background region. The $y_{i}\in \mathbb{R}$ are then generated as distinct nonlinear functions of $x_{i}$ for each cluster (the first row of Figure 3).

It can be seen that in this more challenging simulation, only GPSC is able to recover the true functional clusters, with the results of each clustering algorithm plotted in the spatial domain in Figure 3 (see Supplement D.2 for more details).

### 4.3. Simulation 3 - Model Robustness

In Simulation 3, we present a more realistic scenario of three clusters that have some degree of spatial separation. Motivated by our real-world application of clustering North Carolina census tracts, the sun and moon clusters could be interpreted to represent two urban centers surrounded by a larger rural region. By applying the spatially penalized version of GPSC, we will show that the clustering results remain stable across both increasing levels of noise, as well as to overspecification of the input number of clusters. Full visualization and comparisons can be found in Supplement D.3, D.4 and D.5.

After generating the data ${\left\{\left(s_{i},x_{i}\right)\right\}}_{i=1}^{n}$ from independent uniform distributions, the spatial domain is subdivided into the three clusters, the sun and moon shape, and the background region. The $y_{i}\in \mathbb{R}$ are then generated as distinct nonlinear functions of $x_{i}$ for each cluster with varying degrees of zero-mean Gaussian noise. For an extension of Simulation 3 to nonlinear functions of both $s_{i}$ and $x_{i}$, see Supplement D.5.

#### Noisy Responses

**Fig. 4:**
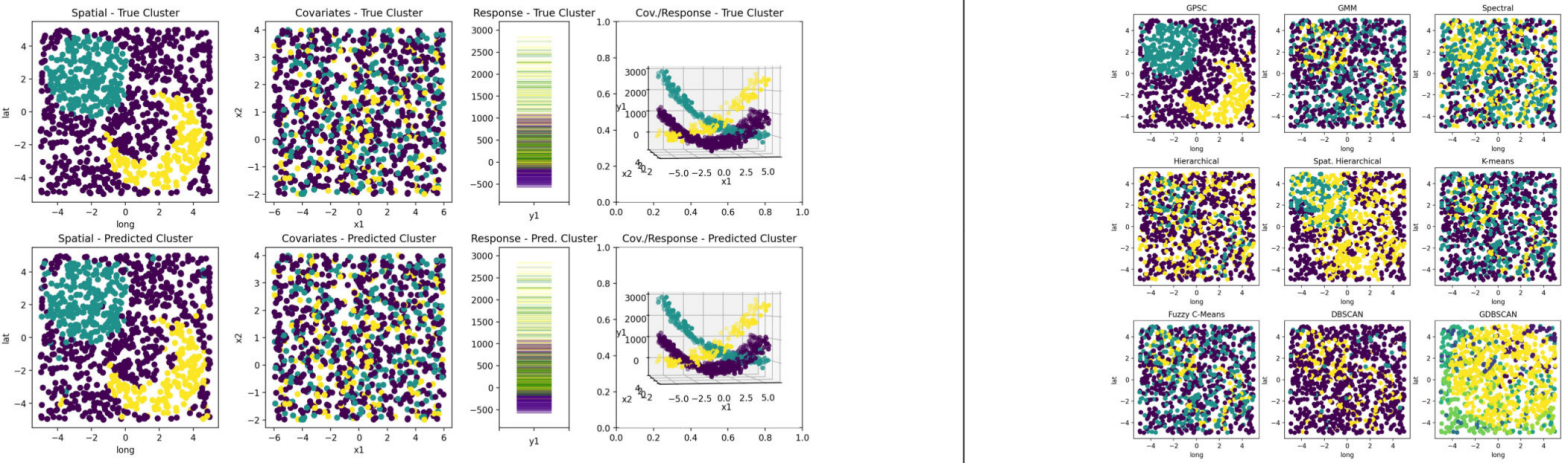
[Left] Results for Simulation 3 with true generated data (top) and results of GPSC (bottom). [Right] Clusters by nine different algorithms visualized in the spatial domain.

We first show that GPSC works under noisy conditions as per Theorem 3.4. In Figure 4, we present Simulation 3 with noise variance = 100, showing that the spatially penalized version of GPSC still performs well under noisy conditions. In particular, GPSC is able to outperform competitors at all tested noise levels, where no other competitor is able to recover the true clusters (with exact ARI/AMI scores and additional details in Supplement D.3).

#### Overspecified Number of Clusters

**Fig. 5:**
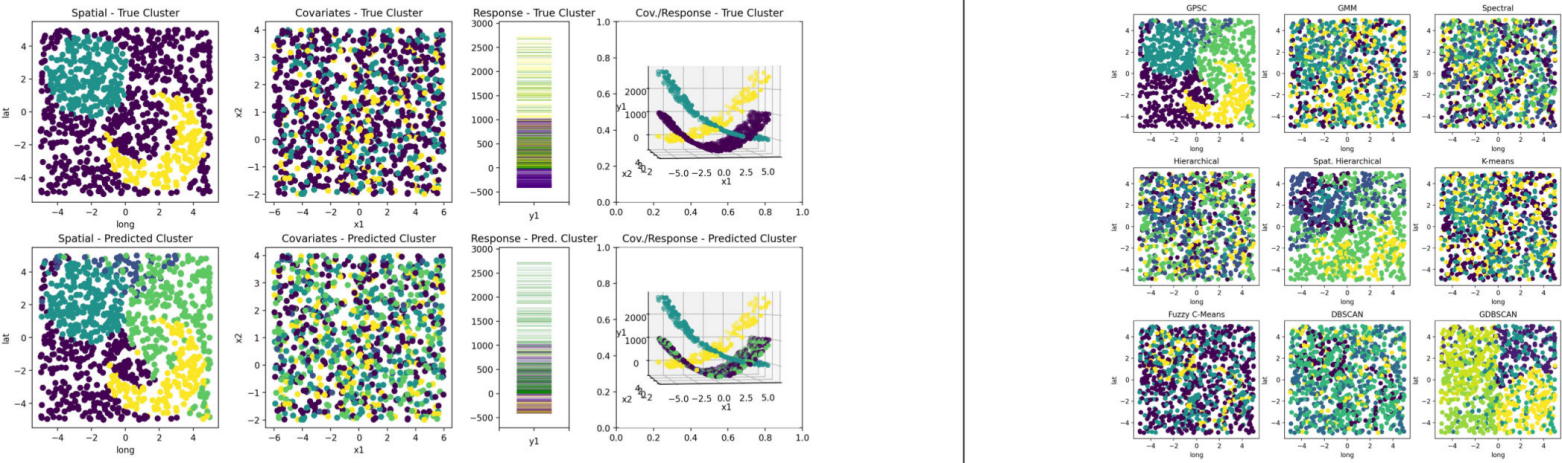
[Left] GPSC results for Simulation 3 with overspecified number of clusters as 5. [Right] Results of nine algorithms with overspecified input presented in the spatial domain.

Finally, we show that GPSC is stable when the number of clusters is overspecified. Specifically, it can be seen in Figure 5 when the number of specified clusters is 5, the sun (teal) and moon (yellow) clusters remain stable, while the background cluster (originally purple) is split into three purple, indigo, and light green clusters. In contrast, the competitors are unable to recover the true clusters when the number of clusters are overspecified, while further visualizations and comparisons to the competitor models are presented in Supplement D.4.

## 5. Applications to NC Tract Data

This dataset consists of 29 community-wide covariates aggregated by census tracts in North Carolina. Such covariates ranged from measures of environmental pollution to averages of socioeconomic indicators such as unemployment, housing environment, education, etc (see Supplement E for a full list). Each census tract is associated with a single (longitude, latitude) pair of coordinates. The overall socioeconomic indicators were previously aggregated using latent class analysis into a single advantage/disadvantage class with 8 categories.^2^

**Fig. 6:**
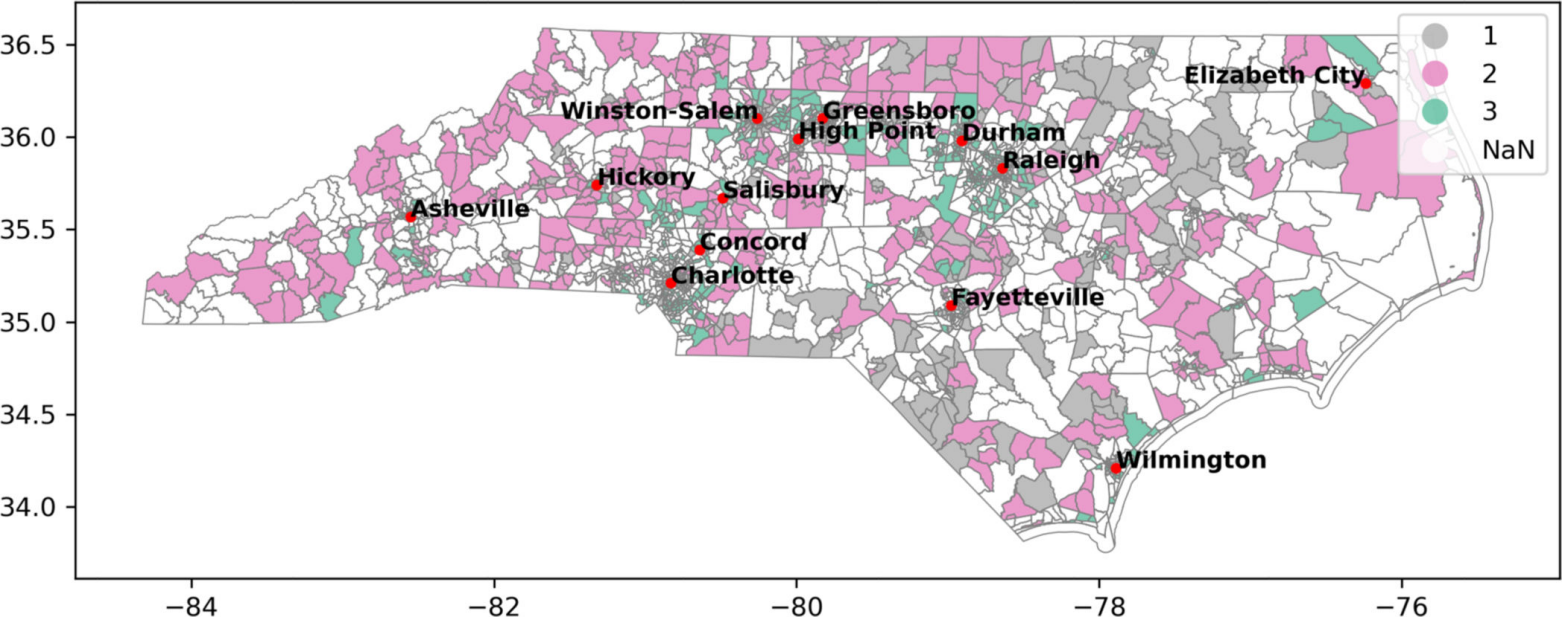
Baseline aggregate groups of socioeconomic and environmental latent class indicator.

Based on the distribution of the full latent classes seen in Figure 1, we can see that there is some degree of separation in the spatial domain between certain groups. Thus, we initialized our GPSC algorithm by performing traditional K-means clustering on solely the spatial domain. We then applied our GPSC algorithm using this latent class as the response variable, taking all other features as the set of covariates. Here, we focus on K-means clustering for comparison due to its interpretable results from previous studies,^2^ with results from other clustering algorithms presented in Supplement E. Based on our results, we find that *L* = 3 produced the most interpretable clusters, and thus aggregated the 8 latent classes into 3 as a baseline against GPSC seen in Figure 6. Using the language of Larson et al. (2020)^2^ for our predicted 3 clusters, we will consider the overall socioeconomic and environmental advantage to be three levels: low (pink), medium (gray), and high (green).

**Fig. 8:**
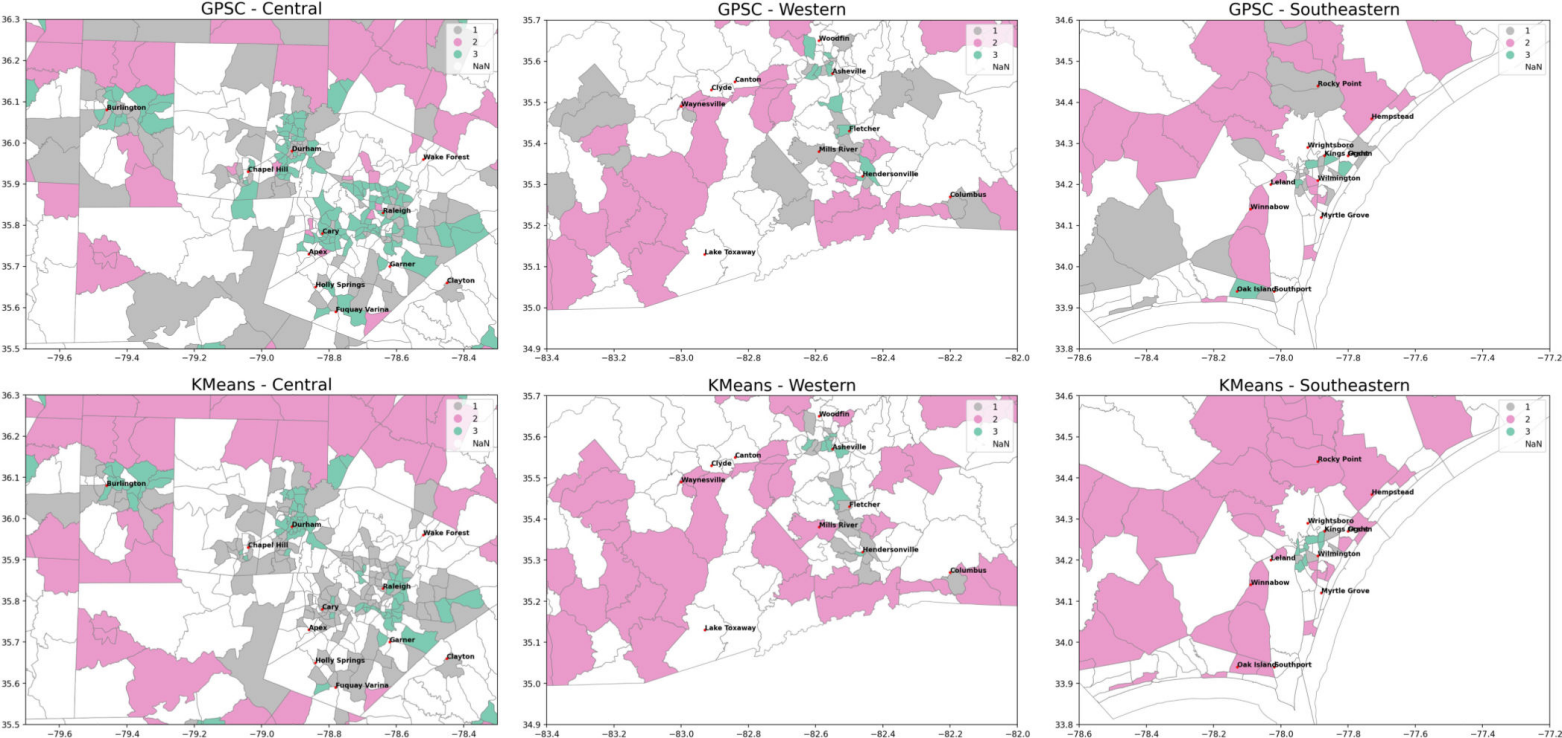
GPSC and K-means cluster results for NC tracts. Column 1: Central NC; Column 2: Western NC (Asheville); Column 3: Southeastern NC (Wilmington)

At first glance, the general spatial distribution of our GPSC and K-means algorithms tends to agree. However, the GPSC predicted clusters differ from K-means and baseline in several meaningful ways. First, in the central region depicted in the first row of Figure 8, GPSC identifies more areas of high advantage (green). Notably, this includes the area surrounding cities such as Chapel Hill, Cary, and the capital city Raleigh (Research Triangle Park), as well as Greensboro and High Point (the Piedmont Triad), which are known to be wealthier and more urbanized regions of the state, whereas the K-means algorithm puts tracts within this region in the medium (gray) advantage group.

Towards the edges of the state we can also see significant differences as the GPSC algorithm tends to further differentiate tracts around the extremities between low and medium advantage. Most notably, around Asheville and Wilmington, two more prominent cities in North Carolina, we are able to distinguish further differences between low and medium advantage tracts, as seen in the second and third rows in Figures 8. Considering the ARI and AMI scores between the two clusterings, we find the scores to be both 0.002, suggesting that clusterings, despite visually seeming to separate the tracts spatially in similar patterns, are actually very different. One challenge of K-means clustering when determining the original 8 latent classes^2^ was a potential lack of finer detail from the K-means predicted clusters. However, here we have shown that despite using the same *L* = 3 clusters, GPSC is able to further differentiate between areas of low and medium disadvantage, in less dense areas of the state along the coast and the western region. Furthermore, there is reason to believe that not all 8 classes are necessary to describe the different advantage groups. In the original grouping, the latent class 2 is actually an empty group, as seen in Figure 1. Thus, the results from GPSC in comparison to K-means and baseline suggest that the algorithm is able to better balance nuance against a traditional clustering algorithm, while also retaining simpler interpretability by using fewer clusters.

## 6. Discussion

Spatial clustering offers unique challenges in comparison to traditional clustering problems due to the spatial domain inherent to geographic data. In our application, the census tract data have distinctly different properties compared to the measured covariates over the tracts. In this paper, we propose a GP-based clustering algorithm and demonstrate its performance in both simulation studies and a real data application. The advantages of GPSC include being able to capture the relative effects between the spatial domain and the measured covariates, largely independent of intersections in the covariate domain as long as the clustered functions themselves have some degree of separation. We also provide theoretical guarantees to the convergence of GPSC and extend it to noisy settings.

GPSC can also be highly scalable; the complexity of the algorithm stems from the fitting of each GP in each iteration, where standard Gaussian processes regression is $O\left(n^{3}\right)$ in the size of the input. In our case, we applied a standard Gaussian process regression model from the scikit-learn package^27^ since our sample size was relatively small. However, in cases of large sample size, scalable GP methods can be applied for a reduction in runtime to O(nlogn).^28^ The GPSC model also has few tuning parameters, notably the number of clusters, optional spatial penalty for data thought to contain spatially contiguous clusters, and and can also be highly flexible through the choice of GP kernel. Although the form of our theorem is independent of the specific choice of kernel (only the convergence rate will differ), in practice more nuanced anisotropic or nonstationary kernels may be more suitable for datasets with strong heterogeneity, for which the actual design of such kernels remains an open problem.

In the real-world application, we applied GPSC to a North Carolina socioeconomic and environmental indicator dataset and found distinct patterns of advantage-disadvantage across the state that captured finer details around the less dense outer regions of the state in comparison to K-means and other clustering methods (presented in Supplement E), while our method also offered simpler interpretability than previous analysis. When utilized by domain experts, the goal of the results of these models is to supplement the identification of marginalized communities, which could be targeted with interventions. Furthermore, in context of our long-term goal of designing interventions, ensuring the accuracy of these models is also of high ethical importance. Therefore in our case, before any application, we can perform sensitivity analyses that tile the geographic region with alternative regional classifiers (county, AHEC region, latitude and longitude tiles of uniform size) to confirm that the same areas arise in multiple boundary definitions. This will confirm that the boundary definitions are not driving artifactual associations. More broadly, it is important that in these high-stakes applications we do not over-rely on any one method. We envisage the possibility of using these clustering results (and GPSC in general) as a supplementary tool for experts to potentially better identify marginalized communities and areas that may be otherwise overlooked.

## Supplementary Material

Supplementary_9789819807024_0025

## Figures and Tables

**Fig. 7:**
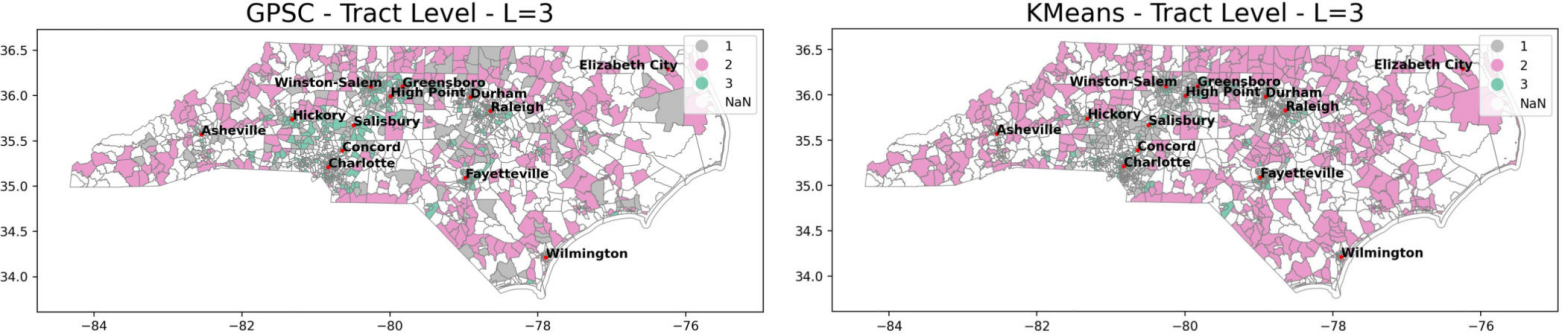
Clusters by GPSC and K-means for tract data, interpreted as overall socioeconomic and environmental advantage between levels of low (pink), medium (grey), and high (green).
