# Supplementary material for: Spatial Clustering for Carolina Breast Cancer Study
Source: Pac Symp Biocomput. Author manuscript; Available in PMC 2026 Jan 3. (PMC12764386; doi:10.1142/9789819807024_0025)
Supplement: Supplementary_9789819807024_0025 [file NIHMS2038215-supplement-Supplementary_9789819807024_0025.pdf]

Supplement for "Spatial Clustering for Carolina Breast Cancer Study"

Hongqian Niu[1], Melissa Troester[2], and Didong Li[1,†]

[1]Department of Biostatistics, [2]Department of Epidemiology, University of North Carolina, Chapel Hill, NC, USA. †E-mail: didongli@unc.edu

A. Supplement Overview

In Section B we continue the discussion section of the main paper on the potential limitations of our algorithm and provide some general guidance on usage. In Section C we provide proofs for the two convergence theorems presented in the main paper. Section D contains the full implementation details, and additional figures for the three simulation studies presented in the main paper, as well as an extension to spatially-dependent functions. Section E contains additional details for the real-world application of the main paper as well as additional comparisons to competitor algorithms.

B. Potential Limitations

Here we continue our discussion from the paper on potential limitations of the GPSC model. The first is the question of tuning the appropriate number of clusters. This is a well-known challenge in clustering, which is beyond the scope of our study. However, some clustering algorithms have the capability to automatically determine the number of clusters. In this regard, we found that DBSCAN and GDBSCAN generally performed poorly in our simulation studies, resulting in incorrect and irrelevant clusters. As such, like in our real world application, selecting the appropriate number of clusters for the problem is best handled on a case-by-case basis with input from domain experts or prior background knowledge. The same can be said of the optional tuning parameter λ, which again reinforces contiguous spatial restraints by penalizing assignments to distant clusters. In our application, we were able to compare our results with previous studies on the socioeconomic and environmental cancer risk factors across the state, as well as collaborate with epidemiologists and cancer experts familiar with the datasets.

Next we briefly revisit the modeling assumptions of GPSC. Our main focus is spatial clustering, as motivated by the CBCS application, where different spatial clusters exhibit different functional relations between the response variable y and input features. In this case, according to our theorems presented in the main paper, the performance of GPSC is influenced by several key factors, including D_u, D_l, E_u, and E_l. In simpler terms, if the true underlying functions f_j in different clusters are not clearly distinguishable, or they have unbounded derivatives, it may be challenging to achieve optimal clustering results. However, this is a

common challenge for most clustering algorithms, and overcoming this limitation may require more advanced techniques and designs.

C. Proofs

C.1. *Proof of Theorem 3.3*

We first consider the case of $L = 2$ that is, there are two clusters. Let l_i be the unobserved true cluster label of the x_i and $\widehat{l}_i$ be the cluster label of x_i in the current iteration. Let x_0 be a sample to be clustered with (unobserved) label $l_0 = 1$, that is, x_0 should be assigned to cluster-1. Our goal is to show that GPSC does assign x_0 to cluster-1 under the condition explicitly stated in Theorem 3.3.

Let $(X_1, Y_1) := \{(x_i, y_i) : \widehat{l}_i = 1\}$ be the set of samples assigned to cluster-1 with size $n_1 := \#\{i : \widehat{l}_i = 1\}$. Similarly, let $(X_2, Y_2) := \{(x_i, y_i) : \widehat{l}_i = 2\}$ be the set of samples assigned to cluster-2 with size $n_2 := \#\{i : \widehat{l}_i = 2\}$. According to Algorithm 1, we train two GPR models based on (X_1, Y_1) and (X_2, Y_2), to obtain two predictors of y_0 denoted by $\widehat{y}^{(1)}$ and $\widehat{y}^{(2)}$. Under the notation in Definition 3.2, we have $\widehat{y}^{(1)} = \psi_{X,x_0}(Y_1)$ and $\widehat{y}^{(2)} = \psi_{X,x_0}(Y_2)$, it suffices to show that $e_1 := |y_0 - \widehat{y}^{(1)}| < e_2 := |y_0 - \widehat{y}^{(2)}|$ as long as

$$\frac{n_{21}}{n_{22}} < \frac{D_l E_l}{D_u E_u} - \frac{\|f\|e^{-c_1 n_1^{\frac{1}{p}}} + \|f\|e^{-c_2 n_2^{\frac{1}{p}}}}{D_u E_u}.$$

To calculate e_1, we introduce the following partially observed dummy variables $\widetilde{Y}_1 := f_1(X_1)$ and let $\widetilde{y}^{(1)} := \psi_{X_1,x_0}(\widetilde{Y}_1)$. We plug this term in e_1 and apply triangle inequality to obtain the following:

$$e_1 = |y_0 - \widehat{y}^{(1)}| = |y_0 - \widetilde{y}^{(1)} + \widetilde{y}^{(1)} - \widehat{y}^{(1)}| \le \underbrace{|y_0 - \widetilde{y}^{(1)}|}_{①} + \underbrace{|\widetilde{y}^{(1)} - \widehat{y}^{(1)}|}_{②}.$$

Observe that ① is the prediction error of standard Gaussian process regression on $(X_1, \widetilde{Y}_1)$, without any misspecified samples. As a result, the upper bound of ① comes from Lemma C.1, the asymptotic theory of Gaussian process regression. That is, ① $\le \|f\|e^{-c_1/h_{n_1}}$ for some constant c_1. Assumption (A1) and Dudley's theorem imply that $h_{n_1} = O(n_1^{-\frac{1}{p}})$, so ① $\le \|f\|e^{-c_1 n_1^{\frac{1}{p}}}$.

To analyze ②, we first observe that $\widehat{y}^{(1)} = \psi_{X_1,x_0}(Y_1)$ is based on partially correct clusters, while $\widetilde{y}^{(1)} = \psi_{X_1,x_0}(\widetilde{Y}_1)$ is based on true clusters. Then by the differentiability of ψ, we have

$$② = |\psi_{X_1,x_0}(Y_1) - \psi_{X_1,x_0}(\widetilde{Y}_1)| \le \|\nabla\psi_{X_1,x_0}\|_\infty \|Y_1 - \widetilde{Y}_1\| = D_u\|Y_1 - \widetilde{Y}_1\|,$$

where $D_u = \sup_{X_1 \subset X, x_0 \in X} \|\nabla\psi_{X_1,x_0}\|_\infty$. As a result, it suffices to find an upper bound of $\|Y_1 - \widetilde{Y}_1\|$.

Observe that among samples in (X_1, Y_1), some are correctly clustered, denoted by $(X_{11}, Y_{11}) = \{(x_i, y_i) : l_i = 1, \widehat{l}_i = 1\}$ with size n_{11}, while the rest are incorrectly clustered, denoted by $(X_{21}, Y_{21}) = \{(x_i, y_i) : l_i = 2, \widehat{l}_i = 1\}$ with size n_{21}. After reordering the samples, we have $X_1 = \begin{bmatrix} X_{11} \\ X_{21} \end{bmatrix}$ and $Y_1 = \begin{bmatrix} Y_{11} \\ Y_{21} \end{bmatrix}$. By the model assumption, for the correctly clustered samples

$Y_{11} = f_1(X_{11})$, while for the incorrectly clustered samples, $Y_{21} = f_2(X_{21}) \neq f_1(X_{21})$. By the same rule, we can split $\widetilde{Y}_1$ into two components as well, i.e., $\widetilde{Y}_1 = \begin{bmatrix} \widetilde{Y}_{11} \\ \widetilde{Y}_{21} \end{bmatrix}$ with $\widetilde{Y}_{11} = f_1(X_{11}) = Y_{11}$ and $\widetilde{Y}_{21} = f_1(X_{21})$. That is, the difference between Y_1 and $\widetilde{Y}$ only comes from Y_{21} and $\widetilde{Y}_{21}$:

$$|Y_1 - \widetilde{Y}_1\| = \left\| \begin{bmatrix} Y_{11} \\ Y_{21} \end{bmatrix} - \begin{bmatrix} \widetilde{Y}_{11} \\ \widetilde{Y}_{21} \end{bmatrix} \right\| = \left\| \begin{bmatrix} f_1(X_{11}) \\ f_2(X_{21}) \end{bmatrix} - \begin{bmatrix} f_1(X_{11}) \\ f_1(X_{21}) \end{bmatrix} \right\|$$
$$= \|f_2(X_{21}) - f_1(X_{21})\| \leq n_{21}\|f_2 - f_1\|_\infty = n_{21}E_u,$$

where $E_u = \|f_2 - f_1\|_\infty$. Combining ① and ②, we derive the upper bound of e_1:

$$e_1 \leq C_1 e^{-c_1 n_1^{\frac{1}{p}}} + n_{21} D_u E_u.$$

Then we calculate e_2 by similar idea, but with all inequalities reversed. Again, we introduce the partially unobserved variables $\widetilde{Y}_2 := f_1(X_2)$ and let $\widetilde{y}^{(2)} := \psi_{X_2,x_0}(\widetilde{Y}_2)$. Again, by triangle inequality, we fin the following lower bound of e_2:

$$e_2 = |y_0 - \widehat{y}^{(2)}| = |y_0 - \widetilde{y}^{(2)} + \widetilde{y}^{(2)} - \widehat{y}^{(2)}| \geq \underbrace{|\widetilde{y}^{(2)} - \widehat{y}^{(2)}|}_{③} - \underbrace{|y_0 - \widetilde{y}^{(2)}|}_{④}$$

Finding the upper bound for ④ follows similar logic as for the upper bound for ①. Observe that ④ is the prediction error of standard Gaussian process regression on $(X_2, \widetilde{Y}_2)$, without any misspecified samples. As a result, the upper bound of ④ comes from Lemma C.1 and Assumption (A1). That is, ④ $\leq \|f\| e^{-c_2 n_2^{\frac{1}{p}}}$ for some constant c_2.

While, unlike finding upper bound for ②, our goal is to find a lower bound for ③. By mean value theorem,

$$③ = |\psi_{X_2,x_0}(\widetilde{Y}_2) - \psi_{X_2,x_0}(Y_2)| \geq \inf \|\nabla\psi_{X_2,x_0}(Y)\|_\infty \|Y_1 - \widetilde{Y}_1\| = D_l\|\widetilde{Y}_2 - Y_2\|.$$

To find the lower bound of $\|\widetilde{Y}_2 - Y_2\|$, we again split both vectors into two components: $X_2 = \begin{bmatrix} X_{12} \\ X_{22} \end{bmatrix}$ and $Y_2 = \begin{bmatrix} Y_{12} \\ Y_{22} \end{bmatrix}$, where $Y_{12} = f_2(X_{12})$ and $Y_{22} = f_1(X_{22})$. Then,

$$\|Y_2 - \widetilde{Y}_2\| = \left\| \begin{bmatrix} Y_{12} \\ Y_{22} \end{bmatrix} - \begin{bmatrix} \widetilde{Y}_{12} \\ \widetilde{Y}_{22} \end{bmatrix} \right\| = \left\| \begin{bmatrix} f_1(X_{12}) \\ f_2(X_{22}) \end{bmatrix} - \begin{bmatrix} f_1(X_{12}) \\ f_1(X_{22}) \end{bmatrix} \right\|$$
$$= \|f_2(X_{22}) - f_1(X_{22})\| \geq n_{22} \inf_{x\in\Omega} |f_2(x) - f_1(x)| \geq n_{22}E_l.$$

Combining ③ and ④, we find the lower bound of e_2:

$$e_2 \geq n_{22} D_l E_l - C_2 e^{-c_2 n_2^{\frac{1}{p}}}.$$

Finally, we conclude that $e_1 < e_2$ if $C_1 e^{-c_1 n_1^{\frac{1}{p}}} + n_{21} D_u E_u < n_{22} D_l E_l - C_2 e^{-c_2 n_2^{\frac{1}{p}}}$, that is inequality 1.

Lemma C.1 (wendland2004scattered)**.** *When $f \in \mathcal{N}_K(\Omega)$ where K is the RBF kernel in $\mathbb{R}^p$, then let $\widehat{f}_n$ be the approximation to f by GP based on training samples (X, Y) with sample size n and filled distance $h_n := \sup_{x\in\Omega} \min_i \|x - x_i\|$, then*

$$\|f - \widehat{f}_n\|_\infty \leq e^{-c/h_n}\|f\|_K. \tag{3}$$

To prove Theorem 3.3 for arbitrary L and j, the only difference is in the construction of $\widetilde{Y}_j$, which splits into L components. To analyze e_j, $\widetilde{Y}_j = [\widetilde{Y}_{1j}, \cdots, \widetilde{Y}_{Lj}]^\top$ where $\widetilde{Y}_{kj} = f_j(Y_{kj})$ with $\widetilde{Y}_{jj} = Y_{jj}$. As a result, $\|\widetilde{Y}_j - Y_j\| \le \sum_{k\neq j} n_{kj} D_u E_u$ and

$$e_j \le \|f\| e^{-c_1 n_j^{\frac{1}{p}}} + \sum_{k\neq j} n_{kj} D_u E_u.$$

Similarly, for e_k with $k \neq j$, $\widetilde{Y}_k = [\widetilde{Y}_{1k}, \cdots, \widetilde{Y}_{Lk}]^\top$ where $\widetilde{Y}_{mk} = f_j(Y_{mk})$ with $\widetilde{Y}_{jk} = Y_{jk}$. As a result, $\|\widetilde{Y}_k - Y_k\| \ge \sum_{m\neq k} n_{mk} D_l E_l$ and

$$e_k \ge \sum_{m\neq k} n_{kj} D_l E_l - \|f\| e^{-c_k n_k^{\frac{1}{p}}}.$$

C.2. *Proof of Theorem 3.4*

For simplicity, we show the case of $L = 2$ only, the extension to general case is similar to the proof of Theorem 3.3. Following the proof in Section C.1, it sufficies to analyze ①. Recall that $y_0 = f_1(x_0) + \epsilon$, we first define $y_{0*} := f_1(x_0)$, then $|y_0 - y_{0*}| \le |\epsilon| \le 3\tau$ with probability 99.7% since $\epsilon \sim N(0, \tau^2)$. Since y_{0*} is the clean observation without any noise, the previous analysis carries to $|y_{0*} - \widetilde{y}(1)|$ naturally, that is,

$$\begin{aligned} ① &= |y_0 - \widetilde{y}^{(1)}| = |y_0 - y_{0*} - y_{0*} + \widetilde{y}^{(1)}| \\ &\le |y_0 - y_{0*}| + |y_{0*} - \widetilde{y}^{(1)}| \le |\epsilon_0| + \|f\| e^{-c_1 n_1^{\frac{1}{p}}} \end{aligned}$$

where $\epsilon_0 \sim (0, \tau^2)$. To analyze ②, by the same argument, it suffices to bound $\|Y_1 - \widetilde{Y}_1\|$.

$$\begin{aligned} \|Y_1 - \widetilde{Y}_1\| &= \left\| \begin{bmatrix} Y_{11} \\ Y_{21} \end{bmatrix} - \begin{bmatrix} \widetilde{Y}_{11} \\ \widetilde{Y}_{21} \end{bmatrix} \right\| = \left\| \begin{bmatrix} f_1(X_{11}) \\ f_2(X_{21}) \end{bmatrix} + \Delta - \begin{bmatrix} f_1(X_{11}) \\ f_1(X_{21}) \end{bmatrix} \right\| \\ &= \|f_2(X_{21}) - f_1(X_{21})\| + \|\Delta_1\| \le n_{21}\|f_2 - f_1\|_\infty = n_{21} E_u + \|\Delta_1\|, \end{aligned}$$

where Δ_1 is the vector of noise ϵ's so $\|\Delta_1\| \sim \chi(n_1)$. As a result,

$$e_1 \le C_1 e^{-n_1^{\frac{1}{p}}} + n_{21} D_u E_u + |\epsilon_0| + \|\Delta_1\|.$$

In the same logic, we have ③ $\ge n_{22} D_l E_l - \|\Delta_2\|$, ④ $\le |\epsilon| + \|f\| e^{-c_2 n_2^{-\frac{1}{p}}}$, and

$$e_2 \ge n_{22} D_l E_l - C_2 e^{-n_2^{\frac{1}{p}}} - |\epsilon_0| - \|\Delta_2\|.$$

Finally, we conclude that $e_1 < e_2$ if

$$C_1 e^{-n_1^{\frac{1}{p}}} + n_{21} D_u E_u + |\epsilon_0| + \|\Delta_1\| < n_{22} D_l E_l - C_2 e^{-n_2^{\frac{1}{p}}} - |\epsilon_0| - \|\Delta_2\|,$$

that is,

$$n_{21} D_u E_u < n_{22} D_l E_l - C_1 e^{-c_1 n_1^{\frac{1}{p}}} - C_2 e^{-c_2 n_2^{\frac{1}{p}}} - 2|\epsilon| - \|\Delta_1\| - \|\Delta_2\|.$$

Note that $2|\epsilon| = 2\tau\chi(1)$, $\|\Delta_1\| = \tau\chi(n_1)$ and $\|\Delta_2\| = \tau\chi(n_2)$. Then Theorem 3.4 follows by setting $\xi = 2|\epsilon| + \|\Delta_1\| + \|\Delta_2\|$, the sum of independent χ-distributions with degrees of freedom $1, n_1$ and n_2 rescaled by 2τ, τ and τ respectively.

The limiting case holds since $\chi(n)/n \xrightarrow{n\to\infty}$, that is, the χ random variable grows sub-linearly with the degree of freedom.

D. Details on Simulation Studies

All simulation experiments were carried out on an Apple Macbook Pro with M1 Pro processor with 32 GB of memory. The scikit-learn clustering package[27] and scikit-fuzzy[29] package were used for all experiments to perform traditional clustering as well as handling Gaussian process regression for the GPSC algorithm and computing clustering metrics. All code for the simulation studies has also been made available. Note that for all simulations in the main paper, as well as in each of the additional simulations presented in this section, GPSC and all other competitor algorithms were tuned on a single random seed. Experiments were then replicated using these same parameters 49 more times on the next 49 random seeds in order for a total of 50 replicates, and the adjusted Rand index and adjusted mutual information scores are reported as mean $\pm$ standard deviation. Finally, an early stopping condition was employed for all experiments. When both the adjusted Rand index and adjusted mutual information both are above 0.90 (exact value can be set by user) on an iteration by iteration basis, the algorithm is thought to have converged and stopped. The exact values for all experiments are presented with the code.

For parameter tuning of the competitor methods, a grid search over the parameters maximizing the adjusted mutual information score against the true labels was performed as followed: 1) For K-means, the default parameters were used in the scikit-learn package. 2) For spectral clustering, the affinity matrix was determined by nearest neighbors, where the number of neighbors was tuned between 1 and 50 in increments of one. 3) For DBSCAN, the eps parameter (maximum distance between two samples in a single neighborhood) was searched between 1 and 100 in increments of one, and the minimum number of samples in a neighborhood was tuned between 1 and 40 in increments of one. 4) For standard hierarchical clustering, the default parameters were used under the ward linkage. 5) For supervised fuzzy C-means, the default arguments in the scikit-fuzzy package were used, except the algorithm was initialized using the response variable y as the labels. 6) For GDBSCAN, the distance thresholds were tuned individually for each simulation. 7) For spatialized hierarchical clustering, the spatial connectivity matrix was determined by k-nearest neighbors, where the number of neighbors was searched between 1 and at least 75 in increments of one, and where the linkage was also varied between the set {average, complete, ward, single}. 8) Finally for the Gaussian mixture model, the default parameters in the scikit-learn package were used. Any auxiliary parameters unspecified here were left as the default values from the packages.

D.1. *Simulation 1*

The data used in this simulation takes the form $\{(s_i, x_i, y_i)\}_{i=1}^n$, where $s_i \in \mathbb{R}^2$, the spatial domain, $x_i \in \mathbb{R}^2$, the covariate domain, and $y_i \in \mathbb{R}$, the response domain, for visualization purposes.

In this simulation, both $s_i \in \mathbb{R}^2$ and $x_i \in \mathbb{R}^2$ are generated from independent uniform distributions, where $s_i \sim \text{Unif}(-5, 5)$ and $x_i \sim \text{Unif}(-3, 3)$ component-wise.

After generating the data $\{(s_i, x_i)\}_{i=1}^n$, where $n = 1000$ samples, the domain square is subdivided into two clusters, the ball shape cluster and the rest region. This is done by subsetting all points $\{(s_i, x_i)\}$ within 2.8 units of the point (0, 0) solely in the spatial domain into cluster 2 (ball), and the remaining points of the background into cluster 1.

For each cluster, y is generated as a linear function of x. For cluster 1, the true function is:

$$y = -x_1.$$

And for cluster 2, the true function is:

$$y = x_1.$$

After the data was generated, GPSC was applied with the following input: 2 clusters, 50 iterations with early stopping, GP input $\{x_i, s_i\}$, constant bounds $(1e^{-15}, 1e^{6})$, length scale bounds $(1e^{6}, 1e^{15})$, input data. For the GP, the RBF kernel was used. Then K-means clustering, spectral clustering, hierarchical clustering with Ward linkage, and DBSCAN were also applied. For spectral clustering, the affinity matrix was generated with using nearest-neighbors set to 11. For DBSCAN, the maximum distance was set to 77, and minimum number of samples set to 3. The full set of $\{x_i, s_i, y_i\}$ as a vector was input into each algorithm along with $L = 2$ clusters where relevant. For supervised C-means clustering, the supervised labels were set according to the y domain with otherwise default parameters. For GDBSCAN, the covariate distance threshold was set to 3, spatial distance set to 13, and minimum set to 0. For the spatialized hierarchical clustering method, the connectivity matrix was specified using k-nearest neighbors using 1 neighbor and ward linkage. Finally, default parameters were used for Gaussian mixture model. These parameters were found by searching over a wide range of values such that the adjusted mutual information was maximized against the true labels. Parameter tuning for all methods, including GPSC, was only done on the first seed (14), and all replicates used the same set of parameters (for seeds 15-63). Any parameters not mentioned were left as default as per the scikit-learn package. The code is provided for full details and implementation.

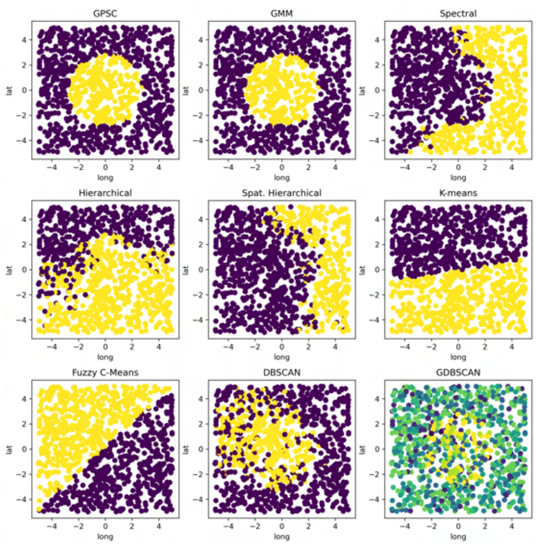

(a) Spatial Domain

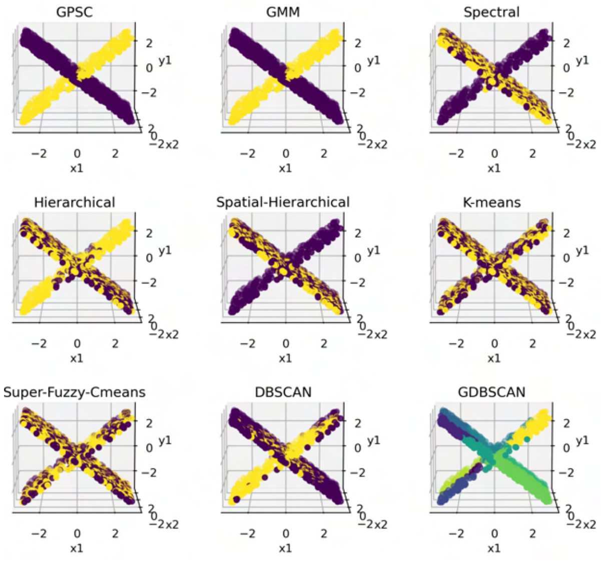

(b) XY Domain

Fig. 9: GPSC and comparisons to spatial clustering and supervised clustering algorithms for Simulation 1.

Table 1: Adjusted Rand index and adjusted mutual information of different methods against the true labels for Simulation 1, replicated over 50 random seeds reported as mean ± standard deviation for.

METHOD	ARI	AMI	METHOD	ARI	AMI
GPSC	$\mathbf{0.91 \pm 0.27}$	$\mathbf{0.90 \pm 0.27}$	GMM	0.82 ± 0.39	0.82 ± 0.39
K-MEANS	0.00 ± 0.00	0.00 ± 0.00	C-MEANS	0.00 ± 0.00	0.00 ± 0.00
HIER.	0.03 ± 0.03	0.11 ± 0.06	SPAT. HIER.	0.03 ± 0.03	0.12 ± 0.06
DBSCAN	0.10 ± 0.08	0.08 ± 0.06	GDBSCAN	0.09 ± 0.03	0.24 ± 0.04
SPECTRAL	0.03 ± 0.14	0.15 ± 0.12			

D.2. *Simulation 2*

The data used in this simulation takes the form $\{(s_i, x_i, y_i)\}_{i=1}^n$, where $s_i \in \mathbb{R}^2$, the spatial domain, $x_i \in \mathbb{R}^2$, the covariate domain, and $y_i \in \mathbb{R}$, the response domain, for visualization purposes.

In this simulation, both $s_i \in \mathbb{R}^2$ and $x_i \in \mathbb{R}^2$ are generated from independent uniform distributions, where $s_i \sim \text{Unif}(-5, 5)$ and $x_i \sim \text{Unif}(-3, 3)$ component-wise.

After generating the data $\{(s_i, x_i)\}_{i=1}^n$, where $n = 1000$ samples, the domain square is subdivided into again two clusters, the ring and background. Cluster 1 (ring) was made by subsetting all points $\{(s_i, x_i)\}$ within 3.5 but greater than 2 units of the point (0,0) solely in the spatial domain, with the rest forming cluster 2.

For each cluster, y is generated as a nonlinear function of just x_i. For cluster 1, the true nonlinear function is:

$$y = -(x_1)^3.$$

And for cluster 2, the true nonlinear function is:

$$y = (x_1)^3.$$

After the data was generated, the GPSC was applied with the following input: 2 clusters, 20 iterations with early stopping, (note that GP input remains $\{x_i, s_i\}$ even though the true functions generating the clusters are functions only of x), constant bounds $(1e^{-15}, 1e^6)$, length scale bounds $(1e^6, 1e^{15})$, input data. For the GP, again the RBF kernel was used.

Then K-means clustering, spectral clustering, hierarchical clustering with Ward linkage, and DBSCAN was also applied. For spectral clustering, the affinity matrix was generated with using nearest-neighbors set to 5. For DBSCAN, the maximum distance was set to 3, and minimum number of samples set to 3. The full set of $\{x_i, s_i, y_i\}$ as a vector was input into each algorithm along with $L = 2$ clusters where relevant. Any parameters not mentioned were left as default as per the scikit-learn package. For spectral clustering, all neighbors between 1 and 50 were tested by comparing the adjusted mutual information scores. For DBSCAN, the maximum distance distance was tested between 1 and 100, and for each distance, the minimum samples were tested between 1 and 300, again by adjusted mutual information scores.

For supervised C-means clustering, the supervised labels were set according to the y domain with otherwise default parameters. For GDBSCAN, the covariate distance threshold was set to 3, spatial distance threshold set to 13, and minimum set to 0. Finally, for the spatialized hierarchical clustering method, the connectivity matrix was specified using k-nearest neighbors using 11 neighbors and ward linkage. For Gaussian mixture model, the default parameters were used. These parameters were found by searching over a wide range of values such that the adjusted mutual information was maximized against the true labels. Parameter tuning for all methods, including GPSC, was only done on the first seed (14), and all replicates used the same set of parameters (for seeds 15-63). Any parameters not mentioned were left as default as per the scikit-learn package. The code is provided for full details and implementation.

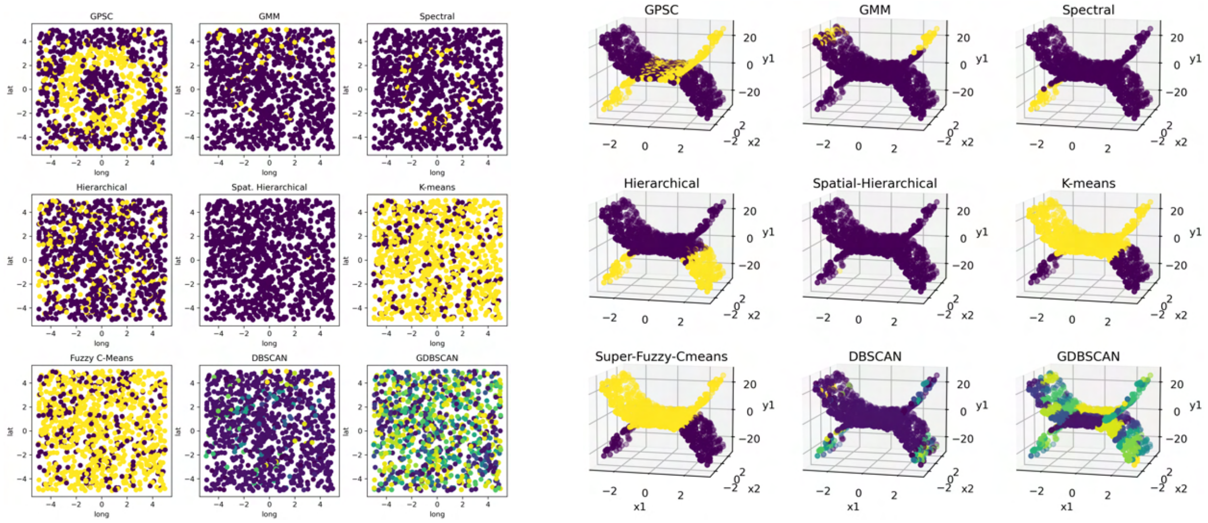

(a) Spatial Domain

(b) XY Domain

Fig. 10: GPSC and comparisons to spatial clustering and supervised clustering algorithms for Simulation 2.

Table 2: Adjusted Rand index and adjusted mutual information of different methods against the true labels for Simulation 2, replicated over 50 random seeds reported as mean ± standard deviation.

METHOD	ARI	AMI	METHOD	ARI	AMI
GPSC	**0.38 ± 0.13**	**0.28 ± 0.10**	GMM	0.11 ± 0.12	0.07 ± 0.11
K-MEANS	0.00 ± 0.01	0.00 ± 0.00	C-MEANS	0.00 ± 0.01	0.00 ± 0.00
HIER.	0.00 ± 0.00	0.00 ± 0.00	SPAT. HIER.	0.00 ± 0.00	0.00 ± 0.00
DBSCAN	0.08 ± 0.05	0.10 ± 0.02	GDBSCAN	0.02 ± 0.01	0.17 ± 0.01
SPECTRAL	0.02 ± 0.09	0.06 ± 0.06			

D.3. *Simulation 3 - Noisy Cluster Results*

The data used in this simulation takes the form $\{(s_i, x_i, y_i)\}_{i=1}^n$, where $s_i \in \mathbb{R}^2$, the spatial domain, $x_i \in \mathbb{R}^2$, the covariate domain, and $y_i \in \mathbb{R}$, the response domain, for visualization purposes. In this simulation, both $s_i \in \mathbb{R}^2$ and $x_i \in \mathbb{R}^2$ are generated from independent uniform distributions, where $s_i \sim \text{Unif}(-5, 5)$, $x_1 \sim \text{Unif}(-6, 6)$, $x_2 \sim \text{Unif}(-2, 4)$ component-wise.

After generating the data $\{(s_i, x_i)\}_{i=1}^n$, where $n = 1000$ samples, the domain square is subdivided into three clusters, the sun shape cluster, moon shape cluster, and the rest region. Cluster 1 was made by subsetting all points $\{(s_i, x_i)\}$ within 2.5 units of the point (-2.2, 2.2) solely in the spatial domain. Cluster 2 was made subsetting all points $\{(s_i, x_i)\}$ within 3 units of (1.8, -1.8) and further than 2 units apart from (1, -1), again solely in the spatial domain, with the remaining points forming cluster 3.

For each cluster, y is generated as a function of just x_i with independent Gaussian distributed noise $\epsilon \sim N(0, \sigma^2)$. For cluster 1, the true nonlinear function is:

$$y = 40x_1^2 - 400 + \epsilon.$$

For cluster 2, the true nonlinear function is:

$$y = -(x_1 - 8)^3 + \epsilon.$$

And for cluster 3, the true nonlinear function is:

$$y = (x_1 + 8)^3 - 20 + \epsilon.$$

After the data was generated, the GPSC was applied with the following input: 3 clusters, 40 iterations with early stopping, (note that GP input remains $\{x_i, s_i\}$ even though the true functions generating the clusters are functions only of x), constant bounds $(1e^{-15}, 1e^4)$, length scale bounds $(1e^6, 1e^{15})$, input data. For the GP, again the RBF kernel was used. Note that here, two forms of GPSC were used. First, standard GPSC was performed with results shown in the table. Then, GPSC with $\lambda = 75$ was used, and it was shown that the GPSC model was better able to find the clusters with this spatial penalty.

Then K-means clustering, spectral clustering, hierarchical clustering with Ward linkage, and DBSCAN was also applied. For spectral clustering, the affinity matrix was generated with using nearest-neighbors set to 12. For DBSCAN, the maximum distance was set to 41, and minimum number of samples set to 27. The full set of $\{x_i, s_i, y_i\}$ as a vector was input into each algorithm along with $k = 3$ clusters where relevant. Any parameters not mentioned were left as default as per the scikit-learn package. For supervised C-means clustering, the supervised labels were set according to the y domain with otherwise default parameters. For GDBSCAN, the covariate distance threshold was set to 675, spatial distance threshold set to 5, and minimum set to 0. For the spatialized hierarchical clustering method, the connectivity matrix was specified using k-nearest neighbors using 9 neighbors and ward linkage. Finally for Gaussian mixture model, the default parameters were used. These parameters were found by searching over a wide range of values such that the adjusted mutual information was maximized against the true labels. Parameter tuning for all methods, including GPSC, was only done on the first seed (14), and all replicates used the same set of parameters (for seeds 15-63). Any parameters not mentioned were left as default as per the scikit-learn package.

D.3.1. $\sigma^2 = 2$

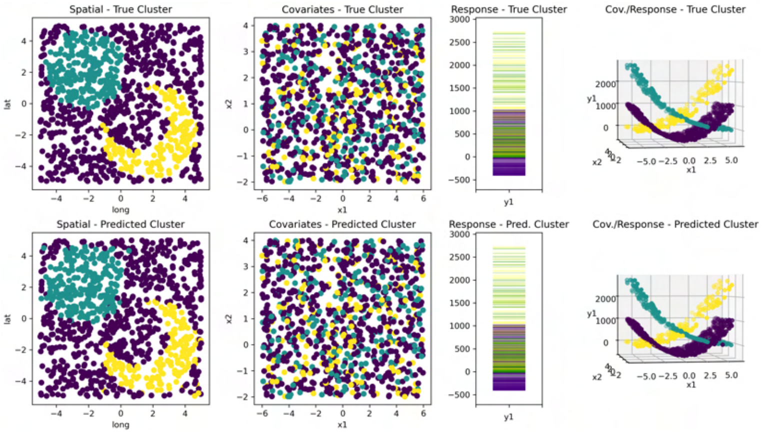

Fig. 11: GPSC results for Simulation 3, $\sigma^2 = 2, L = 3$, colored by cluster and separated by data domain as in previous simulation. The first row indicates ground truth with results from GPSC in the second.

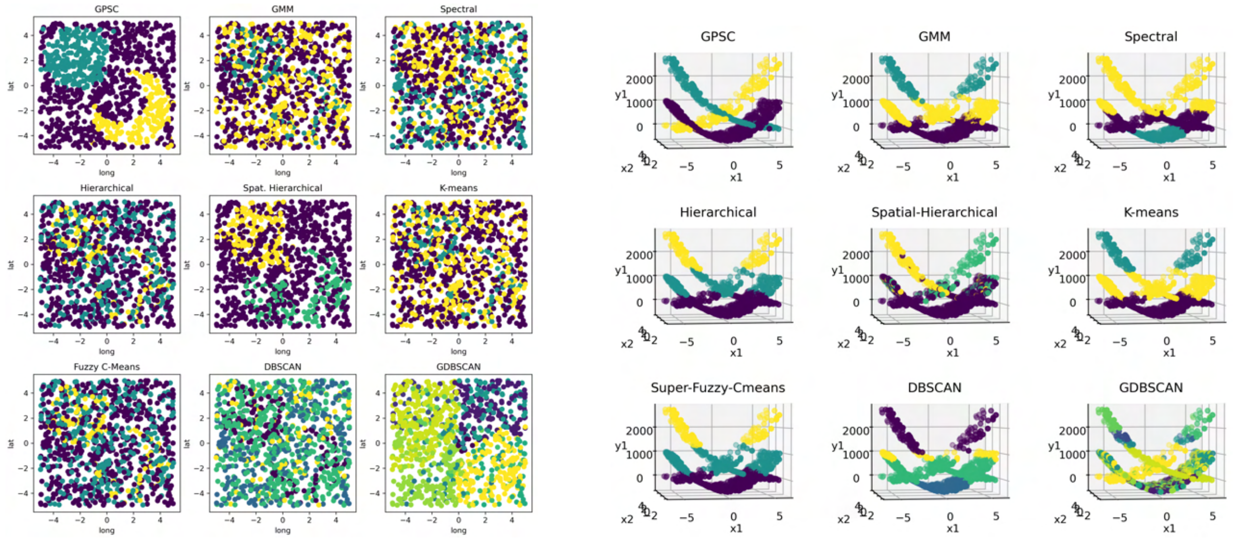

(a) Spatial Domain (b) XY Domain

Fig. 12: GPSC and comparisons to spatial clustering and supervised clustering algorithms for Simulation 3, $\sigma^2 = 2, L = 3$.

Table 3: Adjusted Rand index and adjusted mutual information of different methods against the true labels for for Simulation 3, $\sigma^2 = 2, L = 3$, replicated over 50 random seeds reported as mean $\pm$ standard deviation.

METHOD	ARI	AMI	METHOD	ARI	AMI
GPSC	$\mathbf{0.72 \pm 0.27}$	$\mathbf{0.70 \pm 0.24}$	GMM	0.16 ± 0.02	0.14 ± 0.02
K-MEANS	0.17 ± 0.02	0.13 ± 0.01	C-MEANS	0.16 ± 0.02	0.13 ± 0.01
HIER.	0.17 ± 0.03	0.13 ± 0.03	SPAT. HIER.	0.16 ± 0.11	0.17 ± 0.08
DBSCAN	0.22 ± 0.04	0.15 ± 0.03	GDBSCAN	0.10 ± 0.02	0.24 ± 0.04
SPECTRAL	0.08 ± 0.02	0.16 ± 0.02			

D.3.2. $\sigma^2 = 50$

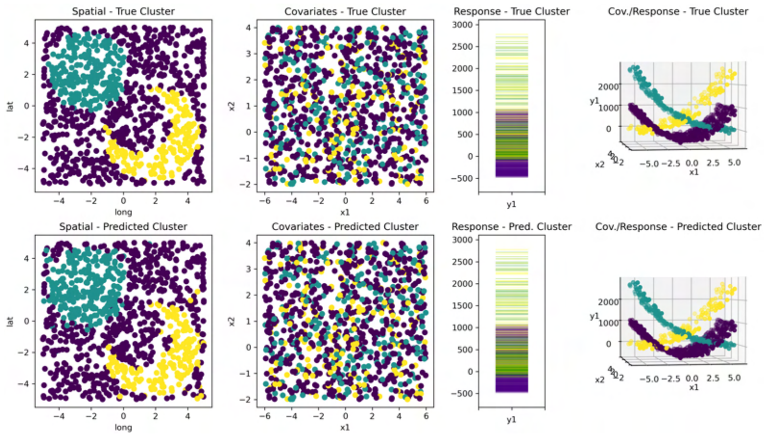

Fig. 13: GPSC results for for Simulation 3, $\sigma^2 = 50, L = 3$, colored by cluster and separated by data domain as in previous simulation. The first row indicates ground truth with results from GPSC in the second.

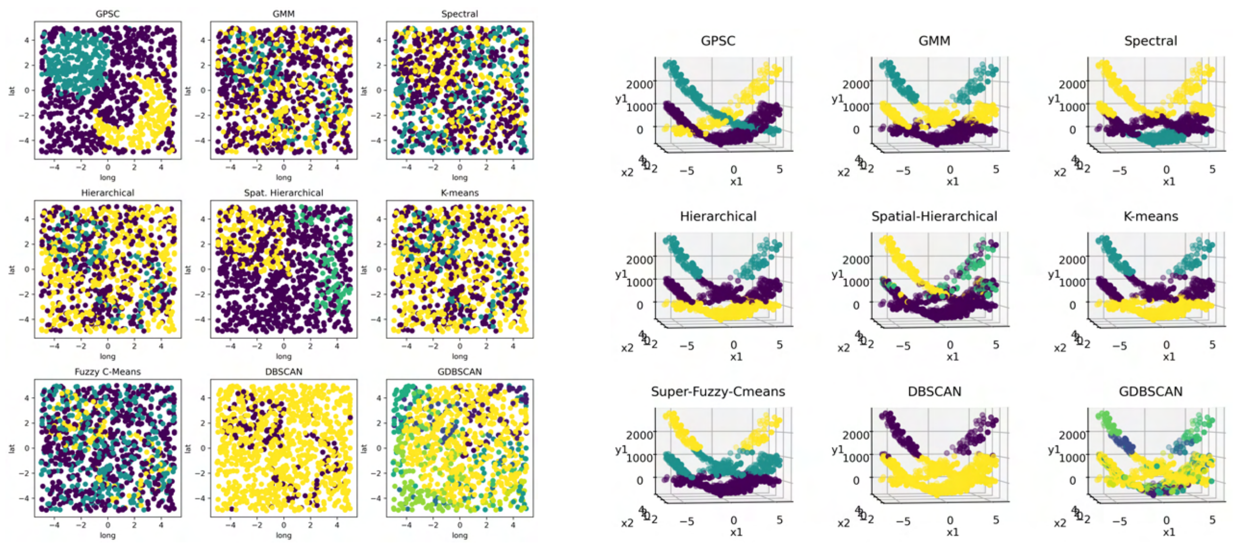

(a) Spatial Domain (b) XY Domain

Fig. 14: GPSC and comparisons to spatial clustering and supervised clustering algorithms for Simulation 3, $\sigma^2 = 50, L = 3$.

Table 4: Adjusted Rand index and adjusted mutual information of different methods against the true labels for for Simulation 3, $\sigma^2 = 50, L = 3$, replicated over 50 random seeds reported as mean ± standard deviation.

METHOD	ARI	AMI	METHOD	ARI	AMI
GPSC	$\mathbf{0.73 \pm 0.25}$	$\mathbf{0.71 \pm 0.22}$	GMM	0.16 ± 0.02	0.13 ± 0.03
K-MEANS	0.17 ± 0.02	0.13 ± 0.01	C-MEANS	0.16 ± 0.02	0.13 ± 0.01
HIER.	0.17 ± 0.03	0.13 ± 0.03	SPAT. HIER.	0.17 ± 0.10	0.18 ± 0.08
DBSCAN	0.23 ± 0.03	0.13 ± 0.03	GDBSCAN	0.09 ± 0.03	0.23 ± 0.03
SPECTRAL	0.08 ± 0.02	0.16 ± 0.01			

D.3.3. $\sigma^2 = 100$

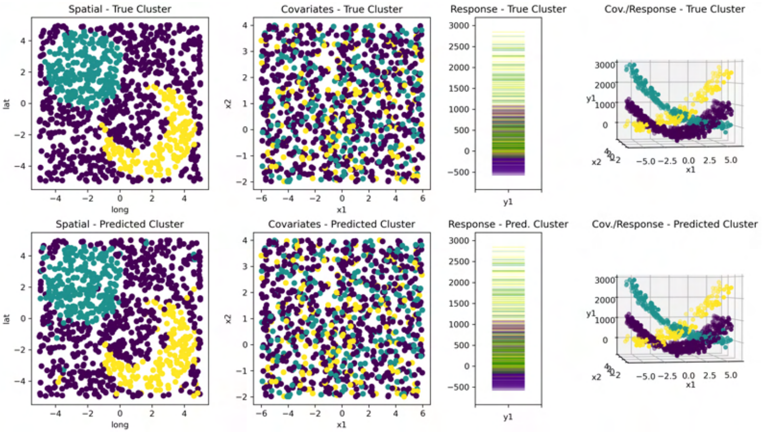

Fig. 15: GPSC results for for Simulation 3, $\sigma^2 = 100, L = 3$, colored by cluster and separated by data domain as in previous simulation. The first row indicates ground truth with results from GPSC in the second.

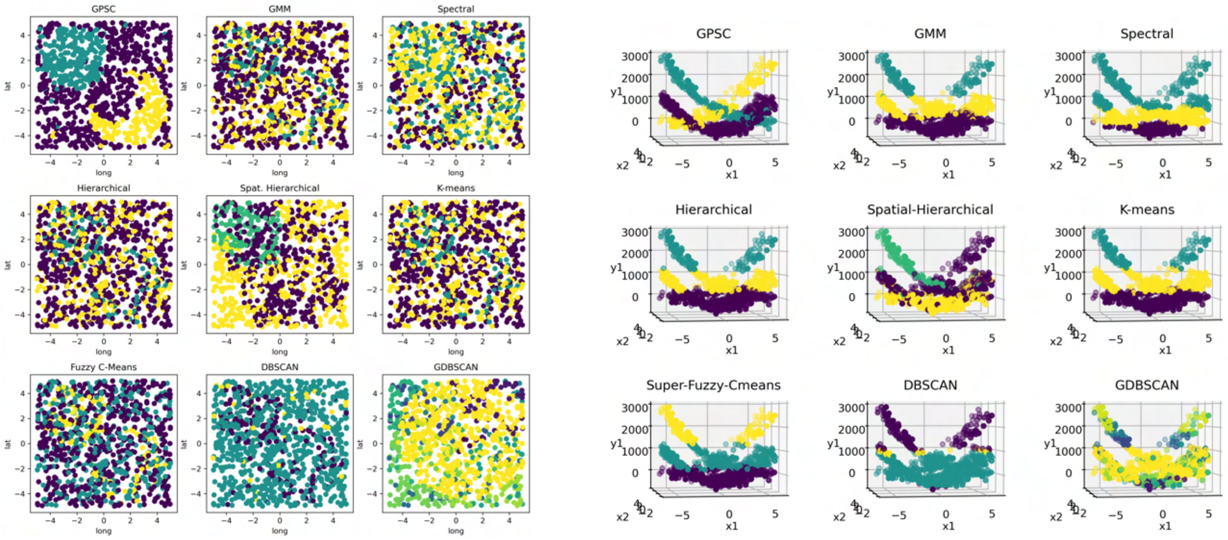

(a) Spatial Domain

(b) XY Domain

Fig. 16: GPSC and comparisons to spatial clustering and supervised clustering algorithms for Simulation 3, $\sigma^2 = 100, L = 3$.

Table 5: Adjusted Rand index and adjusted mutual information of different methods against the true labels for for Simulation 3, $\sigma^2 = 100, L = 3$, replicated over 50 random seeds reported as mean ± standard deviation.

METHOD	ARI	AMI	METHOD	ARI	AMI
GPSC	$\mathbf{0.56 \pm 0.26}$	$\mathbf{0.55 \pm 0.23}$	GMM	0.15 ± 0.02	0.13 ± 0.03
K-MEANS	0.17 ± 0.02	0.13 ± 0.01	C-MEANS	0.16 ± 0.02	0.13 ± 0.01
HIER.	0.16 ± 0.04	0.13 ± 0.03	SPAT. HIER.	0.15 ± 0.09	0.17 ± 0.06
DBSCAN	0.21 ± 0.02	0.10 ± 0.02	GDBSCAN	$0.09 \pm 0.supp03$	0.23 ± 0.04
SPECTRAL	0.07 ± 0.02	0.14 ± 0.01			

D.3.4. $\sigma^2 = 200$

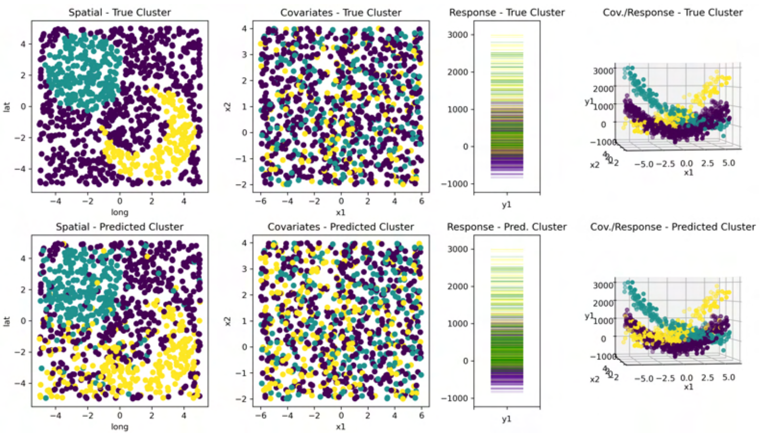

Fig. 17: GPSC results for for Simulation 3, $\sigma^2 = 200, L = 3$, colored by cluster and separated by data domain as in previous simulation. The first row indicates ground truth with results from GPSC in the second.

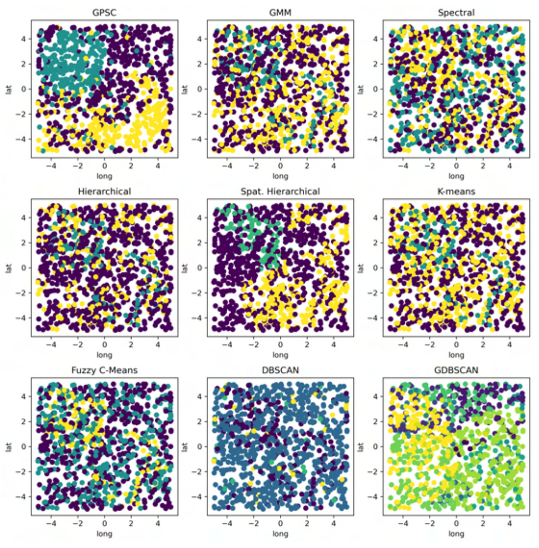

(a) Spatial Domain

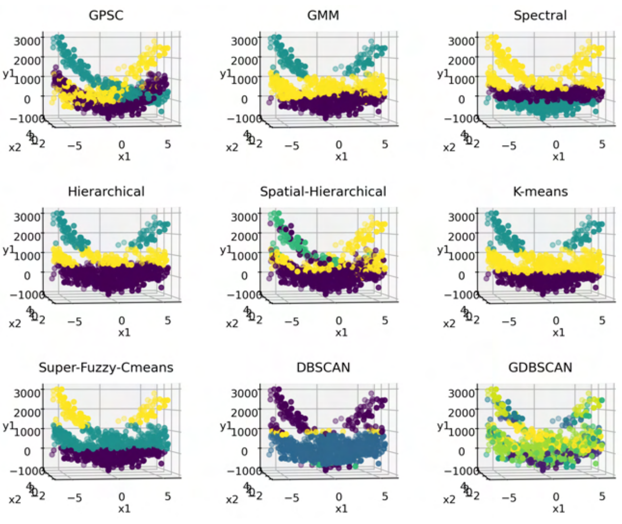

(b) XY Domain

Fig. 18: GPSC and comparisons to spatial clustering and supervised clustering algorithms for Simulation 3, $\sigma^2 = 200, L = 3$.

Table 6: Adjusted Rand index and adjusted mutual information of different methods against the true labels for for Simulation 3, $\sigma^2 = 200, L = 3$, replicated over 50 random seeds reported as mean ± standard deviation.

METHOD	ARI	AMI	METHOD	ARI	AMI
GPSC	$\mathbf{0.33 \pm 0.17}$	$\mathbf{0.33 \pm 0.15}$	GMM	0.16 ± 0.02	0.13 ± 0.02
K-MEANS	0.17 ± 0.02	0.13 ± 0.01	C-MEANS	0.16 ± 0.02	0.13 ± 0.01
HIER.	0.16 ± 0.04	0.12 ± 0.02	SPAT. HIER.	0.15 ± 0.08	0.1 ± 0.06
DBSCAN	0.16 ± 0.02	0.06 ± 0.01	GDBSCAN	0.08 ± 0.02	0.22 ± 0.03
SPECTRAL	0.06 ± 0.02	0.10 ± 0.01			

D.4. *Simulation 3 - Cluster Overspecification Results*

D.4.1. $L = 3, \sigma^2 = 2$

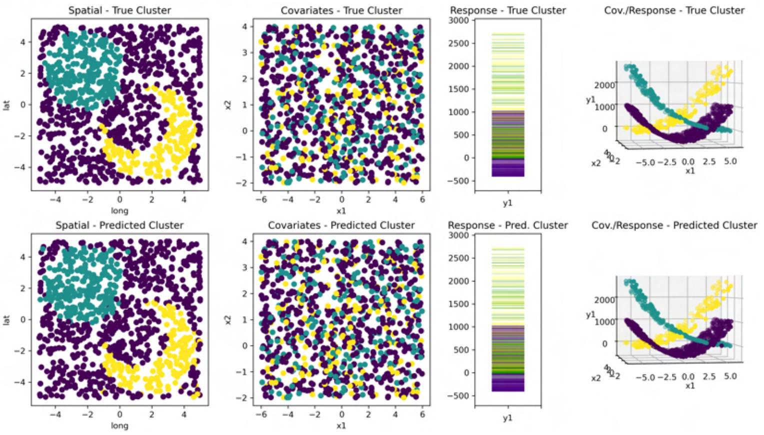

Fig. 19: GPSC results for for Simulation 3, $L = 3, \sigma^2 = 2$, colored by cluster and separated by data domain as in previous simulation. The first row indicates ground truth with results from GPSC in the second.

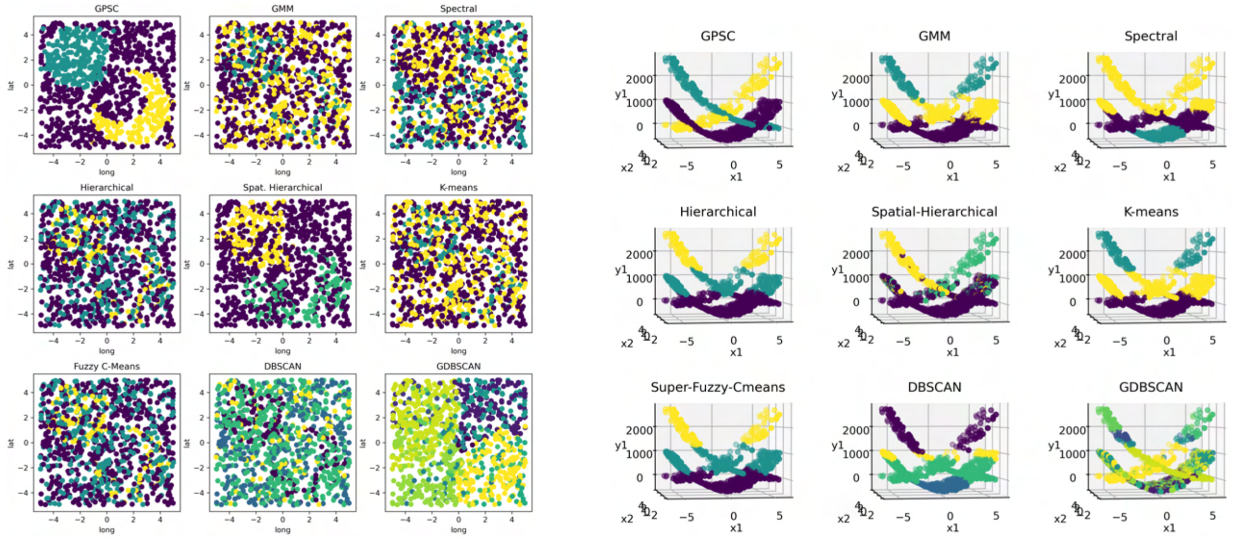

(a) Spatial Domain (b) XY Domain

Fig. 20: Comparisons to spatial and supervised clustering algorithms for Simulation 3, $L = 3, \sigma^2 = 2$.

Table 7: Adjusted Rand index and adjusted mutual information of different methods against the true labels for for Simulation 3, $L = 3, \sigma^2 = 2$, replicated over 50 random seeds reported as mean ± standard deviation.

METHOD	ARI	AMI	METHOD	ARI	AMI
GPSC	**0.72 ± 0.27**	**0.70 ± 0.24**	GMM	0.16 ± 0.02	0.14 ± 0.02
K-MEANS	0.17 ± 0.02	0.13 ± 0.01	C-MEANS	0.16 ± 0.02	0.13 ± 0.01
HIER.	0.17 ± 0.03	0.13 ± 0.03	SPAT. HIER.	0.16 ± 0.11	0.17 ± 0.08
DBSCAN	0.22 ± 0.04	0.15 ± 0.03	GDBSCAN	0.10 ± 0.02	0.24 ± 0.04
SPECTRAL	0.08 ± 0.02	0.16 ± 0.02			

D.4.2. $L = 4, \sigma^2 = 2$

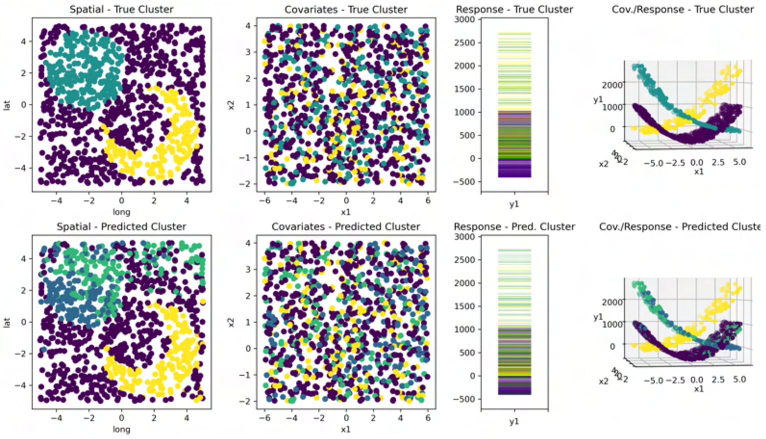

Fig. 21: GPSC results for for Simulation 3, $L = 4, \sigma^2 = 2$, colored by cluster and separated by data domain as in previous simulation. The first row indicates ground truth with results from GPSC in the second.

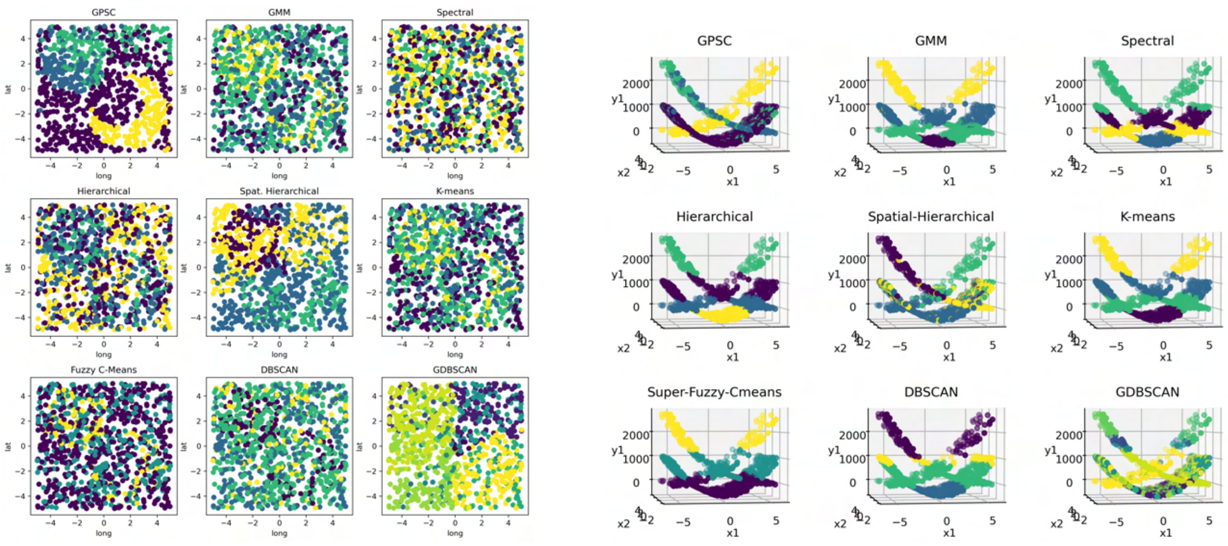

(a) Spatial Domain (b) XY Domain

Fig. 22: GPSC and comparisons to spatial clustering and supervised clustering algorithms, $L = 4, \sigma^2 = 2$.

Table 8: Adjusted Rand index and adjusted mutual information of different methods against the true labels for for Simulation 3, $L = 4, \sigma^2 = 2$, replicated over 50 random seeds reported as mean ± standard deviation.

METHOD	ARI	AMI	METHOD	ARI	AMI
GPSC	$\mathbf{0.58 \pm 0.14}$	$\mathbf{0.69 \pm 0.11}$	GMM	0.08 ± 0.01	0.18 ± 0.02
K-MEANS	0.15 ± 0.02	0.22 ± 0.02	C-MEANS	0.16 ± 0.02	0.13 ± 0.01
HIER.	0.14 ± 0.03	0.19 ± 0.04	SPAT. HIER.	0.13 ± 0.10	0.20 ± 0.07
DBSCAN	0.22 ± 0.04	0.15 ± 0.03	GDBSCAN	0.10 ± 0.02	0.24 ± 0.04
SPECTRAL	0.09 ± 0.01	0.14 ± 0.15			

D.4.3. $L = 5, \sigma^2 = 2$

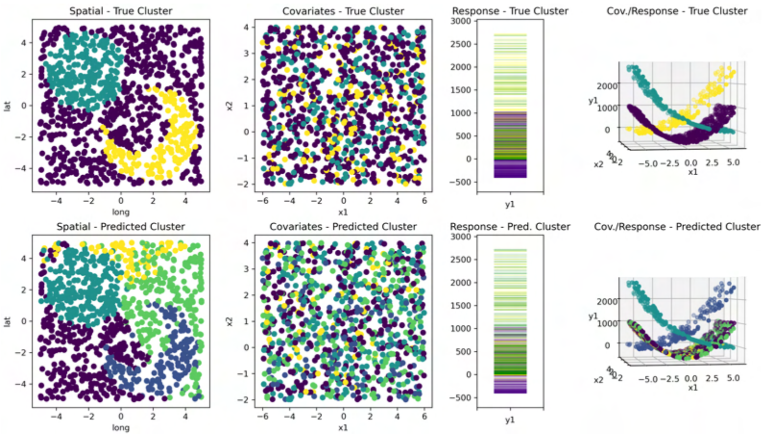

Fig. 23: GPSC results for for Simulation 3, $L = 5, \sigma^2 = 2$, colored by cluster and separated by data domain as in previous simulation. The first row indicates ground truth with results from GPSC in the second.

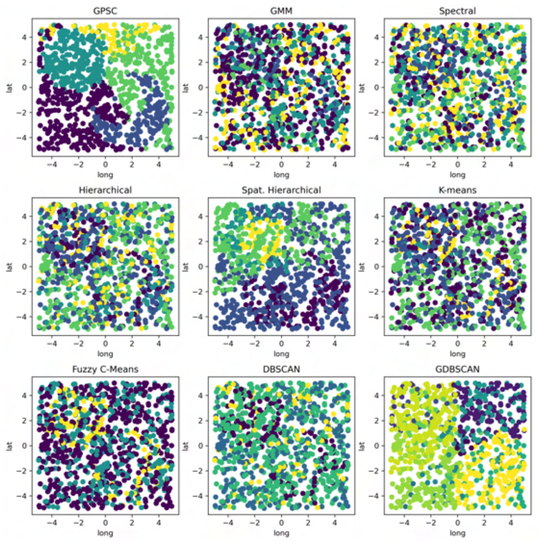

(a) Spatial Domain

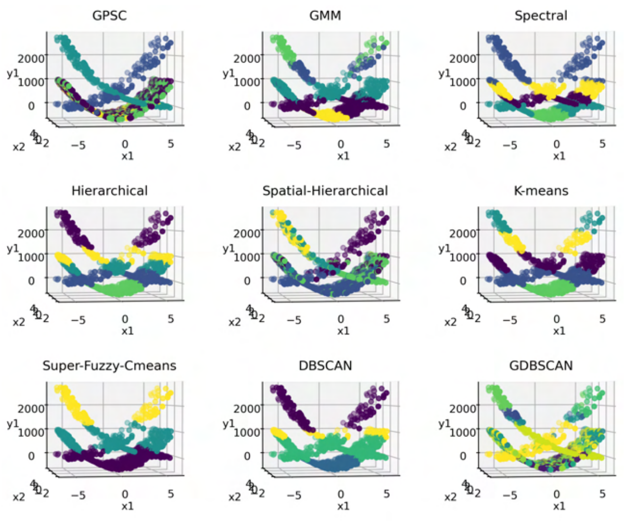

(b) XY Domain

Fig. 24: GPSC and comparisons to spatial clustering and supervised clustering algorithms, $L = 5, \sigma^2 = 2$.

Table 9: Adjusted Rand index and adjusted mutual information of different methods against the true labels for for Simulation 3, $L = 5, \sigma^2 = 2$, replicated over 50 random seeds reported as mean $\pm$ standard deviation.

METHOD	ARI	AMI	METHOD	ARI	AMI
GPSC	$\mathbf{0.47 \pm 0.08}$	$\mathbf{0.65 \pm 0.09}$	GMM	0.10 ± 0.04	0.20 ± 0.06
K-MEANS	0.15 ± 0.02	0.20 ± 0.02	C-MEANS	0.16 ± 0.02	0.13 ± 0.01
HIER.	0.14 ± 0.03	0.20 ± 0.03	SPAT. HIER.	0.13 ± 0.09	0.22 ± 0.06
DBSCAN	0.22 ± 0.04	0.15 ± 0.03	GDBSCAN	0.10 ± 0.02	0.24 ± 0.04
SPECTRAL	0.09 ± 0.01	0.14 ± 0.02			

D.4.4. $L = 6, \sigma^2 = 2$

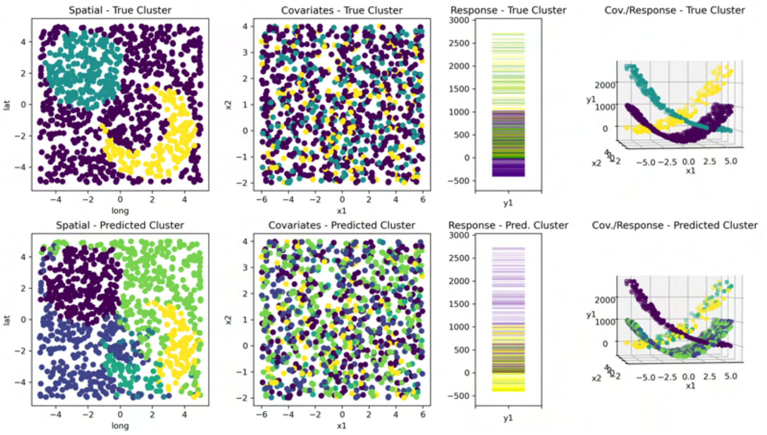

Fig. 25: GPSC results for for Simulation 3, $L = 6, \sigma^2 = 2$, colored by cluster and separated by data domain as in previous simulation. The first row indicates ground truth with results from GPSC in the second.

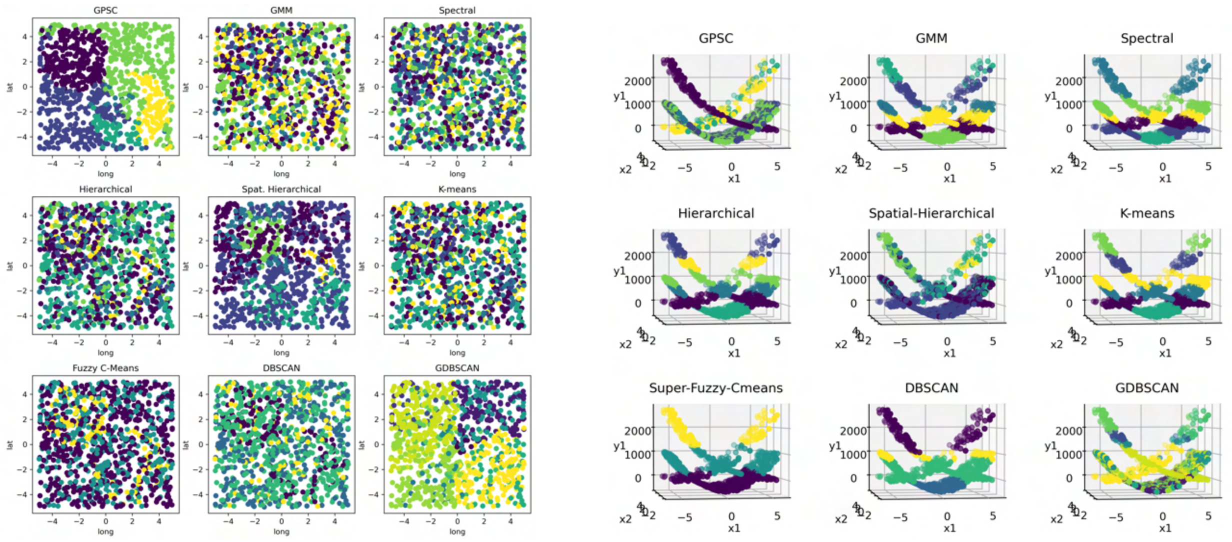

(a) Spatial Domain (b) XY Domain

Fig. 26: GPSC and comparisons to spatial clustering and supervised clustering algorithms for Simulation 3, $L = 6, \sigma^2 = 2$.

Table 10: Adjusted Rand index and adjusted mutual information of different methods against the true labels for for Simulation 3, $L = 6, \sigma^2 = 2$, replicated over 50 random seeds reported as mean $\pm$ standard deviation.

METHOD	ARI	AMI	METHOD	ARI	AMI
GPSC	$\mathbf{0.43 \pm 0.05}$	$\mathbf{0.63 \pm 0.04}$	GMM	0.10 ± 0.03	0.24 ± 0.04
K-MEANS	0.14 ± 0.02	0.19 ± 0.01	C-MEANS	0.16 ± 0.16	0.13 ± 0.01
HIER.	0.14 ± 0.04	0.19 ± 0.02	SPAT. HIER.	0.13 ± 0.08	0.23 ± 0.06
DBSCAN	0.22 ± 0.04	0.15 ± 0.03	GDBSCAN	0.10 ± 0.02	0.24 ± 0.04
SPECTRAL	0.08 ± 0.01	0.18 ± 0.02			

D.5. *Simulation 3 - Functions of s and x*

The set up here is exactly as in Simulation 3, however, the functions are now functions of both the spatial domain and the covariate domain. It can be see that GPSC is still able to recover the true clusters under these conditions. Exact implementation and final parameters can be found in the submitted code.

For each cluster, y is generated as a function of just x_i with independent Gaussian distributed noise $\epsilon \sim N(0, 2)$. For cluster 1, the true nonlinear function is:

$$y = 10(s_1 + s_2)^2 + 40(x_1)^2 + (x_2)^2 - 500 + \epsilon.$$

For cluster 2, the true nonlinear function is:

$$y = 10(s_2)^2 - (x_1 - 8)^3 + (x_2)^3 + \epsilon.$$

For cluster 3, the true nonlinear function is:

$$y = -10(s_1)^2 + (x_1 + 8)^3 + (x_2)^3 - 20 + \epsilon.$$

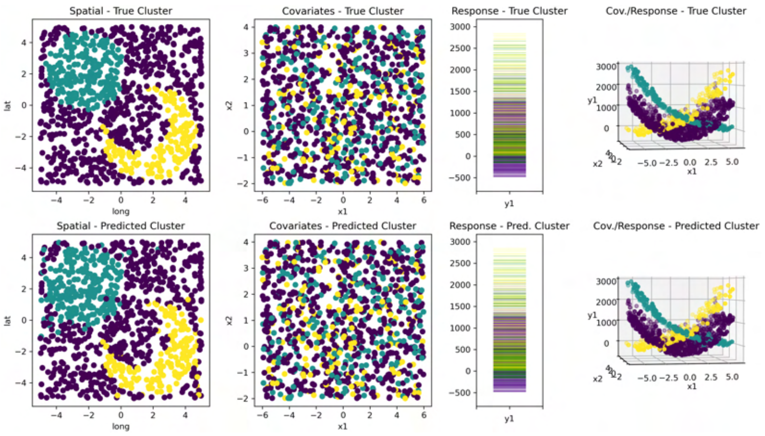

Fig. 27: GPSC results for Simulation 3 analog with functions of both s and x, colored by cluster and separated by data domain as in previous simulation. The first row indicates ground truth with results from GPSC in the second.

As can be seen, GPSC performs well regardless of whether the functional relationships are based on s, x or s and x. Regardless of the which case the true functional relationship is in, the full vector (s, x, y) is used as input and GPSC is able to accurately recover the clusters.

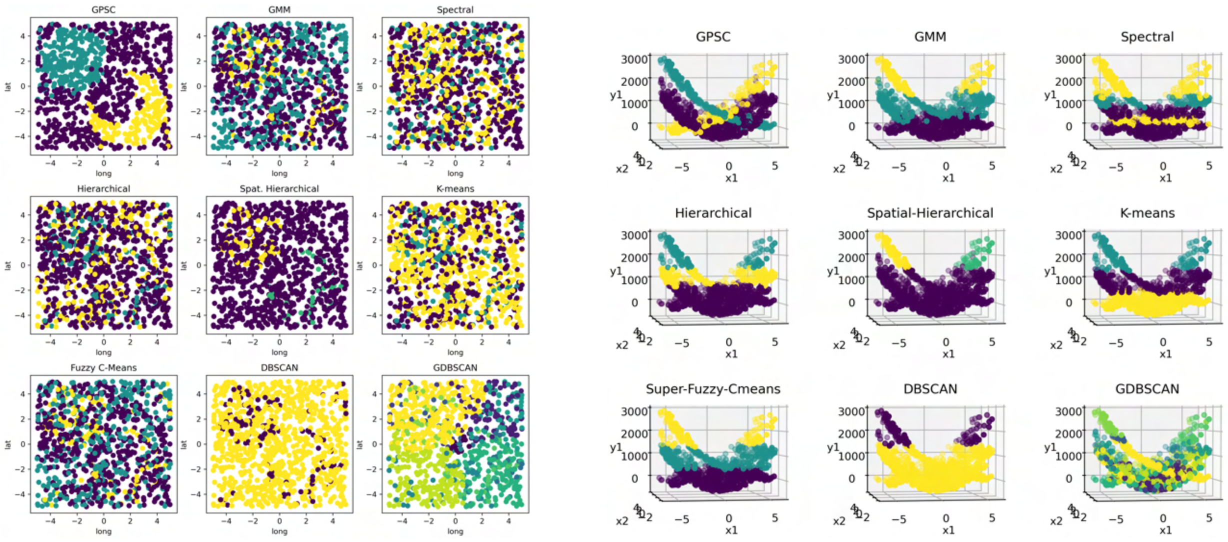

(a) Spatial Domain

(b) XY Domain

Fig. 28: GPSC and comparisons to spatial clustering and supervised clustering algorithms for Simulation 3 analog with functions of both s and x.

Table 11: Adjusted Rand index and adjusted mutual information of different methods against the true labels for Simulation 3 analog with functions of both s and x, replicated over 50 random seeds reported as mean $\pm$ standard deviation.

METHOD	ARI	AMI	METHOD	ARI	AMI
GPSC	$\mathbf{0.69 \pm 0.28}$	$\mathbf{0.65 \pm 0.25}$	GMM	0.09 ± 0.02	0.09 ± 0.02
K-MEANS	0.08 ± 0.01	0.05 ± 0.01	C-MEANS	0.08 ± 0.01	0.05 ± 0.01
HIER.	0.08 ± 0.02	0.05 ± 0.02	SPAT. HIER.	0.10 ± 0.06	0.10 ± 0.07
DBSCAN	0.15 ± 0.02	0.09 ± 0.02	GDBSCAN	0.09 ± 0.02	0.21 ± 0.03
SPECTRAL	0.01 ± 0.05	0.03 ± 0.02			

E. Details on NC Tracts Data

E.1. *Application Details*

The full list of variables used in the clustering analysis is as follows:

Again, the analysis was performed on an Apple Macbook Pro with M1 Pro processor with 32 GB of memory. The scikit-learn clustering package[27] package was used for all experiments to perform comparison K-means clustering as well as handling Gaussian process modeling for the GPSC algorithm and computing clustering metrics. Although we are unable to release the data and auxiliary files for our real world application, the code for clustering and plotting has been submitted along with all the simulation code. For both K-means and GPSC, the full set of data shown in Table 12 including the covariates and spatial data were input into both algorithms.

Variable	Description
**Spatial Data S**	
LATITUDE	Latitude coordinate of population-weighted geographic center of tract
LONGITUDE	Longitude coordinate of population-weighted geographic center of tract
**Covariates X**	
PRFL_M	Men in professional occupation
PRFL_F	Women in professional occupation
LS_HS	Less than high school education
SINGLE	Single with dependent
HSHLDR_F	Female head of household
NHBLK	Non-Hispanic Black
PA	Public assistance
POV	Poverty
NO_VHCL	No vehicle
RENT	Rental housing
CROWD	Crowded housing
UNMPLYD	Unemployment
PHONE	No phone
ACET	Acetaldehyde
BENZENE	Benzene
BUTA	1,3-Butadiene
CARBON	Carbon Tetrachloride
DIESEL	Diesel PM2.5
ETHYL	Ethylbenzene
FORM	Formaldehyde
HEXANE	Hexane
LEAD	Lead compounds
MANG	Manganese compounds
MERC	Mercury compounds
METH	Methanol
METHYL	Methyl Chloride
NICK	Nickel
TOLUENE	Toluene
XYLENE	Xylenes
**Response Y**	
MLCJOINT	Overall class membership into 8 possible groups

Table 12: Full set of variables used for NC tracts data application.

E.2. *Additional Real World Application Comparisons*

In this section we present additional results of the best performing competitors from the simulation studies (using default parameters) on the CBCS real world example of the main paper, which already contained the comparison to K-means clustering. We also include one algorithm of each type from the set of competitors, again for diversity of results. It can be seen that the different types of clustering models have distinct differences to the results of GPSC as discussed below.

E.2.1. *Gaussian Mixture Model*

Here we report the clustering results of the Gaussian Mixture Model. It can be seen that the results are visibly similar to the results of K-means clustering, where again the algorithm appears to mostly center the cluster diversity around the major urban centers of the state, with fewer cluster diversity across the extremities and regions between the urban centers.

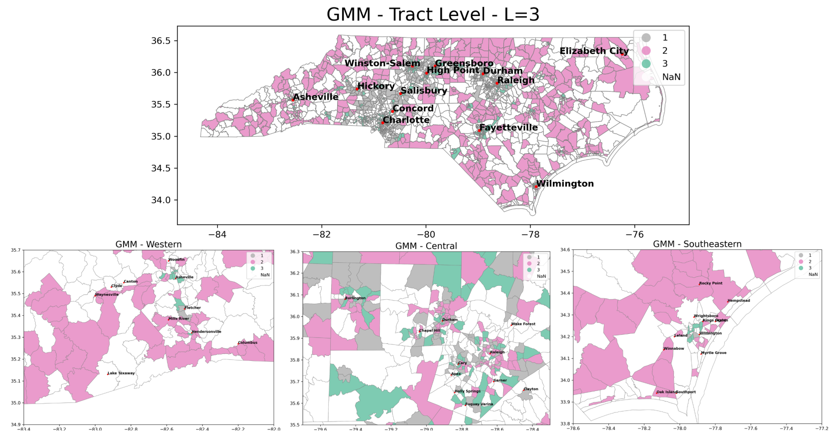

Fig. 29: GMM results for the real world application presented in main paper.

E.2.2. *Spectral Clustering*

Spectral clustering, similar to the spatial hierarchical clustering results presented below, seems to pick up more global trends with lower nuance specifically around the dense city regions of the state.

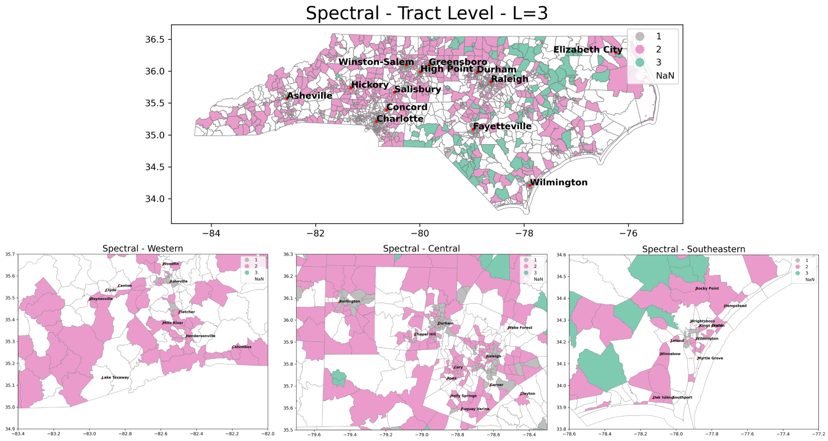

Fig. 30: Spectral clustering results for the real world application presented in main paper.

E.2.3. *Spatial Hierarchical Clustering*

Spatial hierarchical clustering is presented here with 5 neighbors (result did not vary significantly over different specifications of the neighbor count). It can be seen that although the algorithm may be picking up on more global trends across the state, there is decreased nuance around the specific city centers of the state.

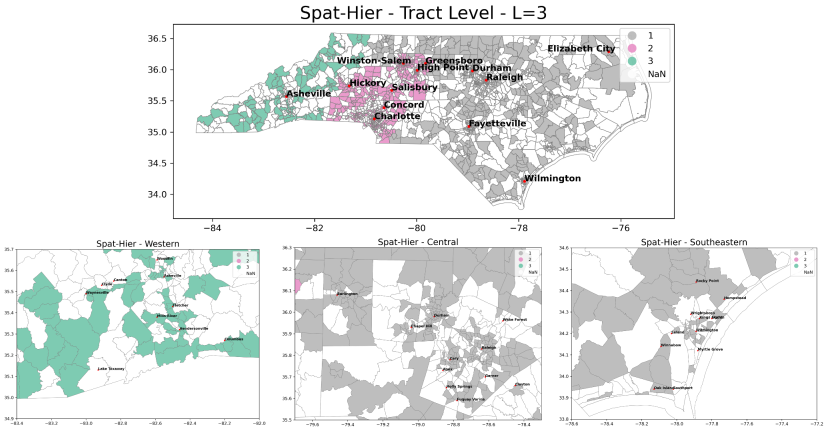

Fig. 31: Spatial hierarchical clustering results for the real world application presented in main paper.

E.2.4. *DBSCAN*

Here DBSCAN was chosen over GDBSCAN due to having fewer hyperparameters required to tune (default used), while having similar performance in the simulation studies. It can be seen here that the main challenge of DBSCAN (as well as GDBSCAN) is the inability to mandate the number of clusters, especially in this application where we specifically seek a small number of clusters for interpretability.

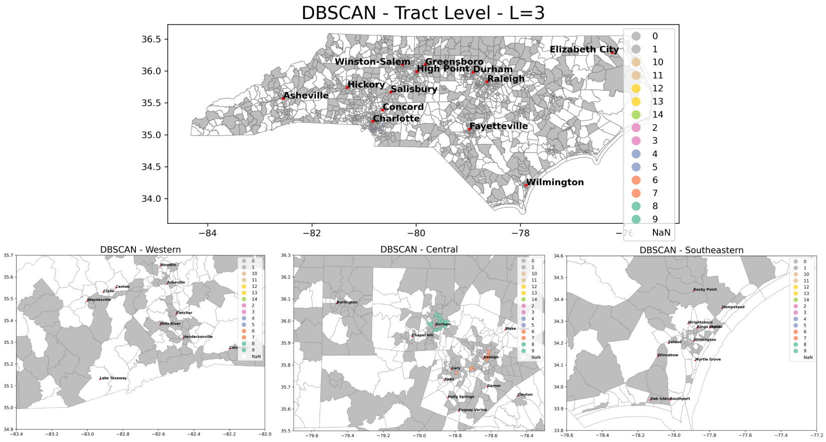

Fig. 32: DBSCAN clustering results for the real world application in the main paper.
